# Impact of preventive primary care on children’s unplanned hospital admissions: a population-based birth cohort study of UK children 2000–2013

**DOI:** 10.1186/s12916-018-1142-3

**Published:** 2018-09-17

**Authors:** Elizabeth Cecil, Alex Bottle, Richard Ma, Dougal S. Hargreaves, Ingrid Wolfe, Arch G. Mainous, Sonia Saxena

**Affiliations:** 1 Department of Primary Care and Public Health, Imperial College London Charing Cross Campus, London, W6 8RP UK; 2 Institute of Child Health, University College London, London, England; 3 Department of Primary Care and Public Health Sciences, King’s College London, London, England; 4 Department of Health Services Research, Management and Policy, University of Florida, Gainesville, FL USA

**Keywords:** Universal health coverage, Child health, Unplanned hospital admissions, Ambulatory care sensitive conditions, Preventive primary care

## Abstract

**Background:**

Universal health coverage (UHC) aims to improve child health through preventive primary care and vaccine coverage. Yet, in many developed countries with UHC, unplanned and ambulatory care sensitive (ACS) hospital admissions in childhood continue to rise. We investigated the relation between preventive primary care and risk of unplanned and ACS admission in children in a high-income country with UHC.

**Methods:**

We followed 319,780 children registered from birth with 363 English practices in Clinical Practice Research Datalink linked to Hospital Episodes Statistics, born between January 2000 and March 2013. We used Cox regression estimating adjusted hazard ratios (HR) to examine subsequent risk of unplanned and ACS hospital admissions in children who received preventive primary care (development checks and vaccinations), compared with those who did not.

**Results:**

Overall, 98% of children had complete vaccinations and 87% had development checks. Unplanned admission rates were 259, 105 and 42 per 1000 child-years in infants (aged < 1 year), preschool (1–4 years) and primary school (5–9 years) children, respectively.

Lack of preventive care was associated with more unplanned admissions. Infants with incomplete vaccination had increased risk for all unplanned admissions (HR 1.89, 1.79–2.00) and vaccine-preventable admissions (HR 4.41, 2.59–7.49). Infants lacking development checks had higher risk for unplanned admission (HR 4.63, 4.55–4.71). These associations persisted across childhood. Children who had higher consulting rates with primary care providers also had higher risk of unplanned admission (preschool children: HR 1.17, 1.17–1.17). One third of all unplanned admissions (62,154/183,530) were for ACS infectious illness. Children with chronic ACS conditions, asthma, diabetes or epilepsy had increased risk of unplanned admission (HR 1.90, 1.77–2.04, HR 11.43, 8.48–15.39, and HR 4.82, 3.93–5.91, respectively). These associations were modified in children who consulted more in primary care.

**Conclusions:**

A high uptake of preventive primary care from birth is associated with fewer unplanned and ACS admissions in children. However, the clustering of poor health, a lack of preventive care uptake, and social deprivation puts some children with comorbid conditions at very high risk of admission. Strengthening immunisation coverage and preventive primary care in countries with poor UHC could potentially significantly reduce the health burden from hospital admission in children.

**Electronic supplementary material:**

The online version of this article (10.1186/s12916-018-1142-3) contains supplementary material, which is available to authorized users.

## Background

Achieving universal health care (UHC) coverage is a Sustainable Development Goal (SDG3.8.1) [1] to improve global health without exposing individuals to financial hardship. Indicators of UHC, such as child immunisation, access to preventive primary care and service capacity [2], are particularly relevant for improving children’s health [3, 4], contributing to substantial progress in reducing under five mortality in children [5].

Yet, even in high-income countries such as the United Kingdom (UK), which has high UHC coverage and where 98% of children are registered with a general practitioner (GP) from birth, children’s health lags behind other Western European nations and unplanned hospital admissions have steadily increased [6, 7]. Rising admissions have been ascribed partly to health system failures to adapt to a growing chronic disease burden in children [6] and primary care policies that have impeded children’s access to high quality primary care [8].

Cross sectional and ecological trend studies using aggregate data have reported correlations between access to primary care and lower hospitalisation rates for ambulatory care sensitive (ACS) conditions in children [7, 8]. ACS conditions in children include common infectious conditions but also chronic conditions commonly seen in primary care (asthma, diabetes and epilepsy) [9, 10].

However, previous studies have been unable to demonstrate how specific UHC interventions such as child immunisation and preventive care around the time of birth may mitigate a child’s risk of admission to hospital, partly due to a previous lack of linked data records between primary care and hospital.

We hypothesised that preventive primary care use, including timely vaccination and development checks, is associated with fewer childhood illness consultations and unplanned and ACS admissions to hospital in a high-income country with a high UHC index.

## Methods

### Study design and data sources

We undertook a birth cohort study using prospectively collected data from the UK Clinical Practice Research Datalink (CPRD), the largest and best validated primary care research database within the UK [11]. It contains longitudinal, patient-level, anonymised computerised health records from 674 general practices and is broadly representative of approximately 7% of the UK population. Clinicians use codes to record diagnoses, prescriptions and procedures, including vaccination. Three quarters (75%) of all English CPRD practices are now linked with Hospital Episode Statistics (HES) [11], which contains International Classification of Diseases version 10 (ICD-10) coded records for the main reason for admission for all National Health Service (NHS) hospitals in England.

### Cohort construction

Our target population was children born between January 1, 2000, and March 31, 2013. A delivery date is provided in the CPRD mother-baby link but also in HES. HES records a child’s birth as an admission method as ‘82’ for a birth of a baby within the hospital or ‘83’ for a baby born outside the hospital [12]. Babies born at home ‘as intended’ will not have a HES birth record. Therefore, to establish a birth cohort we took the child’s date of birth from hospital record and when missing (potentially due to home birth) from the primary care records.

**Fig. 1:**
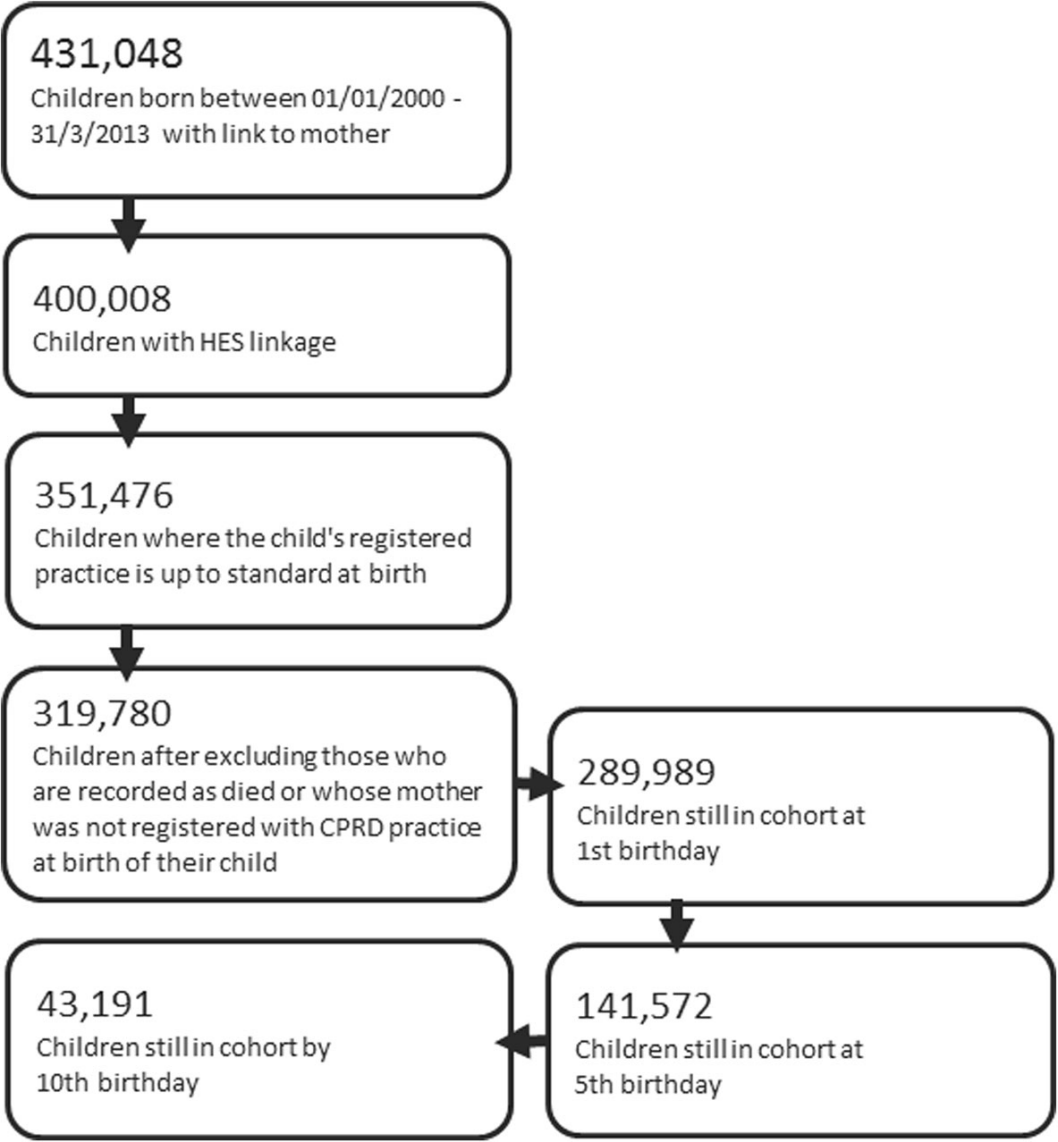
Cohort construction using CPRD participating practice registered children born between January 2000 and March 2013. Children leave the cohort at the end of the study period (31/12/2013). Consequently, children leave the cohort at varying ages, for example, those born in 2013 will only be represented in the cohort’s < 1 year age group. Children may also leave the cohort before the end of the study period if the last practice data collection date was prior to the end of the study period, if they reach their 10th birthday or if they leave their registered practice

To ensure our cohort included children’s full consulting history in primary care from the time of a child’s birth, we included children born to mothers who were registered at an ‘up to standard’ CPRD-participating practice at the time of their birth (Fig. 1). This is because many children are not registered with a GP until their first visit for vaccination and development checks. We assumed that a child would not visit another practice prior to their registration at their mother’s practice.

Children were followed up from birth to the end of the study period (December 31, 2013), the date a child left a practice (deregistered) or the last practice data collection date (whichever came first). During follow-up, children were assigned to one of four developmental age groups, defined as infants (when aged < 1 year), preschool (1–4 years) and primary school (5–9 years). We restricted our cohort to children aged less than 10 years because older children (10–15 years) in CPRD would have had insufficient follow-up time (Fig. 1). We created three sub-cohorts of children aged 5–9 years with ACS conditions as those who had a diagnosis code for asthma, diabetes or epilepsy in their consulting history (Additional file 1: Table S1); this age group was chosen as asthma and epilepsy cannot be reliably diagnosed in young children.

### Exposure to preventive care and illness consultations

In the UK, all children are invited for a development check at 6–8 weeks old (recommended by Healthy Child Programme) and to receive vaccinations at 8, 12 and 16 weeks, and again at 1 and 3 years of age as an integral part of a child’s health and immunisation programme during their early years [13]. At each visit, vaccinations are given both orally and by injection. We determined the dates of vaccinations and also identified children who had incomplete infant vaccinations defined as those with fewer than three consultations for vaccination in their first year and identified children with delayed vaccination if their first vaccine was given after 5 months of age [2].

We defined an infant development check or vaccination as ‘preventive’ consultations (Additional file 1: Table S1) and an illness consultation as any face-to-face clinical contact with a GP excluding those for preventive care [14]. Details of this process are provided in Additional file 2. We explored illness consultations primarily as an explanatory variable for unplanned and ACS admissions.

### Outcomes

The primary outcome was the first record of an unplanned (rather than elective) or ACS admission [7]. We defined admissions for ACS conditions using ICD-10 codes (Additional file 1: Table S2) [9, 10, 15]. ACS infectious illnesses were defined as vaccine-preventable conditions, gastroenteritis, lower and upper respiratory tract infection, and urinary tract infection in all children. We calculated risk for admission with chronic ACS conditions in sub-cohorts of children aged 5–9 years.

Secondary outcomes were illness consultation and unplanned admission rates.

### Covariates: social factors, parenting experience and co-morbid conditions

We examined the relation between several covariates identified from previous studies known to increase admission risk, including social factors and the presence of co-morbid conditions diagnosed in the child’s HES records [16–18].

We identified children who had preterm birth recorded in any diagnosis field of their HES birth record [12] (Additional file 1: Table S3). We also identified children with congenital conditions such as immunodeficiency, cystic fibrosis, chronic lung disease, congenital heart disease, nervous system congenital anomalies, other congenital anomalies (including Down’s syndrome), other perinatal conditions and cerebral palsy (Additional file 1: Table S4) [19].

We assigned each child to one of five population weighted deprivation groups using English Indices of Multiple Deprivation quintiles [20] based on the child’s post code; < 1% of children were missing these data. We identified children of first time mothers to indicate a relative lack of parenting experience compared with mothers who had a previous child, and used maternal age at birth of the child to identify teenage mothers aged < 20 years, who are known to have high consulting rates in primary care, comparing them with mothers aged 20–39 years and older mothers aged 40+ years.

### Statistical analysis

We calculated illness consultation rates by summing consultations for each child divided by their follow-up time in each of the three developmental age bands. We calculated unplanned admission rates as the total number of admissions divided by total follow-up time within each developmental age band. Since unplanned admission rates change with the age of a child, we used Cox proportional hazard model to estimate hazard ratios (HR) for admission. One strength of this methodology is that it does not assume a baseline rate allowing admission rates to change over time. We carried out bivariate and multivariable analyses adjusting (where relevant) for sex, deprivation level, whether the child was a firstborn, maternal age band at birth, child’s prior illness consultation rate (an age band-specific measure: GP consultations prior to admission or, if no admission, within an age band divided by follow-up time), the presence of comorbid health conditions (prematurity and congenital disease), vaccination status and development checks (as preventive care in infants is at 8 weeks old vaccinations and development checks were included as time updating variables when modelling in infants). Covariates were added sequentially to the models based on bivariate association and we considered an association of *p* < 0.05 as statistically significant (model Wald test).

We assessed for interactions between illness consultation and ACS chronic condition status on admission risk. We calculated population attributable risks and, using previously published admission numbers [7], estimated how many preschool children in 2011 could have avoided an admission if fully vaccinated.

We checked Cox proportional hazard assumptions of proportionality, using Nelson–Aalen cumulative hazard plots and non-informative censoring as a sensitivity analysis stratifying by censored versus non-censored children. As sensitivity analyses, we investigated multilevel (random intercept) Cox models, clustering by GP practice. This method models within practice variability allowing GP practice factors such as access to be ignored. We used Stata version 14 (StataCorp, College Station, Texas, USA) for the analyses.

## Results

Our birth cohort consisted of 319,780 children born and registered with 363 English practices between January 2000 and March 2013. One in three of these children (97,836) left their practices during the study period. There were a total of 4,801,171 illness consultations and 183,530 unplanned admissions, with 1,540,977 child-years of follow-up. The median follow-up time (interquartile range) in infants, preschool and primary school children was 1.0 (1.0–1.0), 3.0 (1.1–4.0) and 3.4 (1.5–5.0) years, respectively.

Twenty-three percent (74,233/319,780) of children lived in the most affluent areas compared with 18% (57,440/319,780) of children living in the most deprived areas. Four percent (12,814/319,780) of children were born to teenage mothers (Table 1). One in five infants in the cohort had a record of a congenital condition (18.2%) or were born prematurely (6.0%). Nine percent of primary school children (13,484/141,519) had a diagnosis of an ACS chronic condition (asthma, diabetes mellitus or epilepsy).

**Table 1:** Sociodemographic characteristics and comorbid conditions in birth cohort^a^

Number of children	*N* = 319,780
Characteristics	Number (%)
Boys	163,713 (51.2)
Deprivation^b^	
Least deprived fifth	74,233 (23.2)
Most deprived fifth	57,440 (18.0)
Maternal age at birth of child (years)	
< 20	12,814 (4.0)
20–39	291,846 (91.3)
40+	15,120 (4.7)
Mother’s first child	234,781 (73.6)
Prematurity for constisency	19,275 (6.0)
Congenital condition	57,937 (18.2)
Diagnosed with ACS^c^ chronic condition in children aged 5–9 years (*N* = 141,519)	
Asthma	12,654 (8.9)
Diabetes	268 (0.2)
Epilepsy	670 (0.5)
More than one ACS chronic condition	123 (0.1)

^a^Born between 01/01/2000 and 31/03/2013 registered with 363 practices partnered with the Clinical Practice Research Datalink in England and followed up until 31/12/2013

^b^Index of multiple deprivation fifths (5 the most deprived, 1 least deprived)

^c^Ambulatory care sensitive

Uptake of preventive care was high; 98% (5417/289,989) of preschool children had complete vaccinations, of whom 1% (1736/289,989) had delayed vaccination, and 87% (253,408/289,989) of preschool children had development checks.

Infants had, on average, four illness consultations in their first year; while preschool had 2.9 and primary school aged children had 1.3 illness consultations per year, respectively (Table 2). Primary school children with ACS chronic conditions consulted GPs more frequently than children without, on average, twice per year (Table 2).

**Table 2:** Annual illness consultation in primary care and unplanned and ambulatory care sensitive (ACS) hospital admission rates^a^

Number of children in cohort	Infant (age < 1 year)	Preschool (age 1–4 years)	Primary school (age 5–9 years)	Asthma diagnosis (age 5–9 years)	Diabetes diagnosis (age 5–9 years)	Epilepsy diagnosis (age 5–9 years)
	*N* = 319,780	*N* = 289,989	*N* = 141,572	*N* = 12,654	*N* = 268	*N* = 670
Rate per child/year (95% confidence interval)						
Illness consultation^b^	4.01 (4.00–4.03)	2.91 (2.90–2.92)	1.33 (1.32–1.34)	2.18 (2.15–2.22)	2.02 (1.81–2.23)	2.22 (2.06–2.39)
Rate per 1000 child-years (95% confidence interval)						
Unplanned admissions	259 (256–261)	105 (104–107)	42 (40–44)	84 (77–91)	342 (279–405)	461 (365–558)
Infectious ACS admissions						
URTI	26.6 (25.9–27.2)	19.7 (19.3–20.1)	6.5 (6.2–6.8)			
LRTI	36.1 (35.3–36.9)	4.6 (4.4–4.8)	1.0 (0.9–1.1)			
Gastroenteritis	23.3 (22.7–23.9)	10.5 (10.2–10.7)	2.2 (2.0–2.4)	Not measured	Not measured	Not measured
Urinary tract infection	6.5 (6.1–6.8)	1.9 (1.7–2.0)	0.9 (0.8–1.0)			
Vaccine-preventable infections	0.7 (0.6–0.8)	0.07 (0.06–0.10)	(number too small to compute rate)			
Chronic ACS admissions^c^	N/A	N/A	N/A	26.0 (23.5–28.8)	193 (168–223)	178 (147–216)

^a^Cohort of 319,780 children born between 01/01/2000 and 31/03/2013 registered with 363 Clinical Practice Research Datalink practices linked to Hospital Episode Statistics in England, and followed up until 31/12/2013

^b^Illness consultation: face-to-face consultation with a GP excluding preventive care

^c^ACS chronic admission rates (primary diagnosis at admission) in children aged 5–9 years diagnosed with ACS condition. We chose to analyse the age group alone because asthma cannot reliably be diagnosed in children aged less than 5 years

*ACS* ambulatory care sensitive, *LRTI* lower respiratory tract infection, *URTI* upper respiratory tract infection

### Risk of unplanned hospital admissions

Unplanned admission rates were 259 per 1000 child-years among infants, 105/1000 among preschool children and 42/1000 among primary school children (Table 2). The subset of primary school-aged children diagnosed with an ACS chronic condition (asthma, diabetes and epilepsy) had higher unplanned admission rates compared to all primary school children (84/1000, 342/1000 and 461/1000 child-years for children with ACS chronic conditions).

Lack of preventive care was associated with a higher risk for unplanned admission, after adjusting for deprivation, maternal age and firstborn indicators (Table 3). In preschool children, the adjusted HR for incomplete vaccination was 1.89 (1.79–2.00) and the population attributable risk was 0.0115 (95% CI 0.0101–0.0130). We estimated that, annually, approximately 2000 (0.0115 × 207,573) unplanned admissions in England could be avoided if all preschool children had the same admission rate as those who were fully immunised. In preschool children, those who had delayed vaccinations had an increased risk of unplanned admissions compared to those who had timely vaccinations (HR 1.15, 1.04–1.27). Infants who had no development checks had over four times the risk for unplanned admission than those who had development checks (HR 4.63, 4.55–4.71); this was observed to a lesser extent for preschool and primary school children (HR 1.19 and 1.09, respectively). Illness consultations were associated with a higher risk for unplanned admission, with the risk increasing across childhood; an additional consultation per year increased the average admission rate by 0.5% (HR 1.005, 1.005–1.005) in infants and by 23% (HR 1.23, 1.23–1.24) in primary school children.

**Table 3:** Association of preventive primary care, comorbidity and social factors on risk of unplanned hospital admission^a^

Adjusted hazard ratio^b^ (95% confidence interval)		Infant	Preschool	Primary school
		(age < 1 year)	(age 1–4 years)	(age 5–9 years)
Incomplete vaccinations^c^		1.20 (1.16–1.25)	1.89 (1.79–2.00)	1.27 (1.07–1.51)
No development check^d^		4.63 (4.55–4.71)	1.19 (1.16–1.22)	1.09 (1.03–1.14)
Illness consultation rate^e^		1.00 (1.00–1.00)	1.17 (1.17–1.17)	1.23 (1.23–1.24)
Prematurity		1.21 (1.18–1.25)	1.26 (1.22–1.30)	1.12 (1.04–1.21)
Congenital conditions		2.40 (2.36–2.45)	1.17 (1.15–1.20)	1.15 (1.10–1.21)
Deprivation^f^	5 vs. 1	1.22 (1.20–1.25)	1.38 (1.34–1.41)	1.44 (1.37–1.51)
Maternal age	< 20 vs. 20–39 years	1.33 (1.28–1.38)	1.35 (1.30–1.40)	1.28 (1.18–1.39)
	40+ vs. 20–39 years	0.86 (0.83–0.90)	0.93 (0.89–0.97)	0.96 (0.88–1.05)
Being the firstborn child		0.88 (0.86–0.90)	0.97 (0.95–0.99)	No association
Sex		0.83 (0.81–0.84)	0.87 (0.86–0.89)	0.80 (0.77–0.83)

^a^Cohort of children born between 01/01/2000 and 31/03/2013 registered with 363 practices partnered with the Clinical Practice Research Datalink practices linked to Hospital Episode Statistics in England and followed up until 31/12/2013

^b^Hazard ratios have been adjusted for listed covariates. Covariates were added sequentially to the models, *p* values of < 0.05 were considered statistically significant

^c^In modelling admissions in infants, incomplete vaccination is a time-updated variable. In children aged 1 and over, incomplete vaccination is less than three infant vaccinations

^d^Did not complete infant development checks within primary care

^e^An illness consultation is a face-to-face consultation with a GP which is not for preventive care

^f^Index of multiple deprivation fifths (5 the most deprived, 1 least deprived)

The presence of comorbid conditions and social factors, such as material deprivation, were also strongly associated with unplanned admission. Children born prematurely or with a congenital condition had a greater risk of unplanned admission across all age groups (HR 1.21, 1.18–1.25 in preterm infants; HR 2.40, 2.36–2.45 in infants with congenital conditions). Children living in the most deprived quintile had a 22% higher risk of an unplanned admission from infancy. This increased risk of admission doubled to 44% in primary school years (HR 1.22, 1.20–1.25) and in infants (HR 1.44, 1.37–1.51) (Table 3). Infants of teenage mothers had a 33% increased risk of an unplanned admission compared with older mothers aged 20–39 years HR 1.33 (1.28–1.38).

### Risk factors for ACS infectious admissions (Table 4)

One-third of all unplanned admissions (62,154/183,530) in the birth cohort were for ACS infectious illness, of which only 271 were for vaccine-preventable conditions. Overall, the risk factors for ACS infectious admissions were similar to the risks for all unplanned admissions (Table 4); however, the magnitude of the association between deprivation and ACS infectious illness tended to be greater than for all admissions. Infants with incomplete vaccination were over four times more likely to be admitted for a vaccine-preventable condition (HR 4.41, 2.59–7.49), with the risk persisting, and increasing in magnitude, across childhood.

**Table 4:** Association of preventive primary care, comorbidity and social factors on risk of ambulatory care sensitive infectious admissions^a^

	Infant	Preschool	Primary school
	(aged < 1 year)	(aged 1–4 years)	(aged 5–9 years)
	HR^b^ (95% CI)	HR^b^ (95% CI)	HR^b^ (95% CI)
Vaccine preventable admissions			
Incomplete vaccinations^c^	4.41 (2.59–7.49)	6.62 (2.80 to 15.66)	19.98 (2.40–166.25)
No development check^d^	3.56 (2.64–4.81)	1.95 (3.66–1.04)	No association
Illness consultation rate^e^	1.00 (1.00–1.00)	1.15 (1.10–1.20)	1.26 (1.11–1.43)
Prematurity	No association	No association	No association
Congenital conditions	1.74 (1.26–2.40)	No association	No association
Deprivation^f^ 5 vs. 1	2.11 (1.37–3.25)	2.27 (1.08–4.76)	No association
Upper respiratory tract infection			
Incomplete vaccinations^c^	1.62 (1.40–1.88)	1.58 (1.41–1.77)	No association
No development check^d^	2.16 (2.05–2.27)	1.18 (1.12 to1.24)	1.15 (1.03–1.28)
Illness consultation rate^e^	1.00 (1.00–1.00)	1.16 (1.16–1.17)	1.24 (1.23–1.25)
Prematurity	1.62 (1.50–1.76)	1.32 (1.24–1.40)	1.41 (1.21–1.64)
Congenital conditions	1.39 (1.32–1.48)	1.22 (1.17–1.27)	1.15 (1.03–1.28)
Deprivation^f^ 5 vs. 1	1.54 (1.44–1.65)	1.48 (1.41–1.56)	1.79 (1.60–2.00)
Lower respiratory tract infection			
Incomplete vaccinations^c^	1.53 (1.40–1.68)	2.16 (1.77–2.64)	No association
No development check^d^	2.60 (2.49–2.71)	1.29 (1.17–1.43)	No association
Illness consultation rate^e^	1.00 (1.00–1.00)	1.15 (1.15–1.16)	No association
Prematurity	2.24 (2.11–2.39)	1.87 (1.67–2.10)	1.82 (1.29–2.57)
Congenital conditions	1.36 (1.30–1.43)	1.36 (1.25–1.48)	1.25 (1.23–1.28)
Deprivation^f^ 5 vs. 1	1.50 (1.42–1.60)	1.26 (1.15–1.39)	No association
Gastroenteritis admissions			
Incomplete vaccinations^c^	1.16 (0.99–1.35)	1.82 (1.58–2.09)	No association
No development check^d^	2.02 (1.92–2.13)	1.29 (1.21–1.37)	No association
Illness consultation rate^e^	1.00 (1.00–1.00)	1.15 (1.15–1.16)	1.24 (1.23–1.26)
Prematurity	1.28 (1.17–1.41)	1.19 (1.10–1.30)	1.35 (1.04–1.74)
Congenital conditions	1.40 (1.32–1.49)	1.23 (1.16–1.30)	No association
Deprivation^f^ 5 vs. 1	1.69 (1.58–1.82)	1.67 (1.57–1.78)	1.63 (1.34–1.98)
Urinary tract infection			
Incomplete vaccinations^c^	No association	2.01 (1.43–2.82)	No association
No development check^d^	2.41 (2.17–2.68)	1.28 (1.09–1.50)	No association
Illness consultation rate^e^	1.00 (1.00–1.00)	1.15 (1.14–1.17)	1.27 (1.24–1.30)
Prematurity	1.44 (1.21–1.71)	No association	No association
Congenital conditions	1.35 (1.19–1.52)	1.42 (1.24–1.62)	No association
Deprivation^f^ 5 vs. 1	No association	1.59 (1.35–1.87)	1.42 (1.04–1.95)

^a^Data is from a cohort of 319,780 children born between 01/01/2000 and 31/03/2013, registered with 363 Clinical Practice Research Datalink practices in England, with Hospital Episode Statistics linkage, and followed up until 31/12/2013

^b^Adjusted hazard ratio and 95% confidence interval; hazard ratios have been adjusted for listed covariates as well as sex, maternal age and whether first child; covariates were added sequentially to the models, *p* values of < 0.05 were considered statistically significant

^c^In modelling admissions in infants, incomplete vaccination is a time updated variable; in children aged 1 and over, incomplete vaccination is less than three infant vaccinations

^d^Did not complete infant development checks within primary care

^e^An illness consultation is a face-to-face consultation with a GP that is not for preventive care

^f^Index of multiple deprivation fifths (5 the most deprived, 1 least deprived)

### Risk factors for ACS chronic admissions

Primary school-aged children diagnosed with an ACS chronic condition, compared with children without an ACS chronic condition, had an increased risk of unplanned admission (for any admission diagnosis) of 1.90 (1.77–2.04), 11.43 (8.48–15.39) and 4.82 (3.93–5.91) for asthma, diabetes and epilepsy. There was no evidence that deprivation was a stronger risk factor for children with an ACS chronic condition (Table 5).

**Table 5:** Association of preventive primary care, comorbidity and social factors on risk of ambulatory care sensitive chronic admissions in primary school-aged children

	Primary school
	(aged 5–9 years)
	HR^a^ (95% CI)
Asthma	
Incomplete vaccinations^b^	2.24 (1.11–4.50)
No development check^c^	No association
Illness consultation rate^d^	1.14 (1.11–1.16)
Prematurity	No association
Congenital conditions	No association
Deprivation^e^ 5 vs. 1	1.38 (1.10–1.72)
Diabetes	No Associations
Epilepsy	
Incomplete vaccinations^d^	3.83 (1.21–12.07)
No development check^e^	No association
Illness consultation rate^c^	1.09 (1.05–1.12)
Prematurity	No association
Congenital conditions	No association
Deprivation^e^ 5 vs. 1	No association

^a^Adjusted hazard ratio and 95% confidence interval; hazard ratios have been adjusted for listed covariates as well as sex, maternal age and whether first child; covariates were added sequentially to the models, *p* values of < 0.05 were considered statistically significant

^b^In modelling admissions in infants, incomplete vaccination is a time updated variable; in children aged 1 and over, incomplete vaccination is less than three infant vaccinations

^c^Did not complete infant development checks within primary care

^d^An illness consultation is a face-to-face consultation with a GP that is not for preventive care

^e^Index of multiple deprivation fifths (5 the most deprived, 1 least deprived)

Illness consultation rates modified the association of unplanned admissions for ACS chronic conditions, with interaction factors of 0.92 (0.91–0.93), 0.88 (0.82–0.95) and 0.95 (0.92–0.98) in asthma, diabetes or epilepsy, respectively. For example, a child with asthma has, on average, 90% greater risk of an unplanned admission compared with children without an ACS chronic condition (holding consultation rate constant); this reduced to 74% with a single additional illness consultation per year.

Of the primary school children diagnosed with an ACS chronic condition, 19% (2597/13,484) had an unplanned admission (any diagnoses), while only 5% (729/13,484) had an unplanned admission for an ACS chronic condition.

### Sensitivity analyses

Sensitivity analyses showed that our findings were robust to age and duration of registration. Unplanned admission HRs for variables of interest were similar when children were followed up from 8 weeks of age compared with follow-up from birth. HRs were similar but more extreme in those who were censored due to leaving their registered practice compared with those who remained until the end of the study period (December 2013) or the practice’s last data collection date (if earlier) (Additional file 1: Tables S5 and S6).

Unplanned admission HRs for variables of interest were higher in infants when we applied a multilevel model (Additional file 1: Table S7), but were similar in older age groups.

## Discussion

### Main findings

To our knowledge, this is the largest study of its kind to assess the impact of preventive primary care on the risk of unplanned and ACS hospital admissions across childhood. In our birth cohort of 319,780 children registered with UK primary care and followed over 1,540,977 child-years, there was a high uptake of preventive care, with 98% of children having complete vaccinations and 87% having development checks in infancy and high contact rates with GP (four times in infancy for illness). A lack of preventive primary care, including vaccination and development checks, was strongly associated with a higher risk of unplanned and ACS admission in children. For example, there was a four-fold increased risk of vaccine-preventable hospital admissions that increased across childhood. We estimate that, in the UK, 2000 preschool children are admitted annually to hospital for problems that are potentially attributable to a lack of basic vaccinations. One-third of all unplanned admissions, in our cohort, were for ACS infectious illness. One in five primary school age children who had a chronic ACS condition (asthma, diabetes or epilepsy) were admitted to hospital. We also found comorbidity and social deprivation to be associated with unplanned admission.

### Comparison with past research

We found no previous studies specifically investigating the impact of preventive primary care on children’s admission risk over time. However, our findings are consistent with systematic reviews and numerous other studies that have demonstrated a relation between various features of primary care in adults and avoidable admission [3, 21].

We, and others, have previously reported that emergency department visits and admissions for ACS chronic conditions are associated with poor access to primary care, highlighting the importance of primary care in preventing adverse outcomes for children with chronic conditions [7, 8]. Our study further supports this evidence, which is especially important since the number of children with chronic conditions is increasing. In recent years, adult admission rates for ACS chronic diseases, including asthma, diabetes and epilepsy, are reported to have fallen following major financial incentives in primary care to improve chronic disease management [22], suggesting scope for a similar policy intervention for children.

The age, sex and sociodemographic profile of our sample was representative of the national population [23] and other variable characteristics, such as the proportion of children born to teenage mothers, were comparable over the study period. A similar proportion of our birth cohort was born prematurely compared with national and international estimates [24]. We could not find comparable studies giving estimates of congenital anomalies. One study investigating child mortality found that only 3% of children who died had a congenital anomaly [25]. However, our study purposefully took a much broader definition of congenital conditions, which included comorbid conditions diagnosed within infancy as well as suspected conditions affected by birth.

In England, since 2007/2008, the combined vaccine coverage in infants for diphtheria, tetanus, polio, pertussis and haemophilus influenza B and for pneumonia vaccine has reached 93-95%. Our cohort may have achieved a higher uptake rate firstly because the national picture is likely to include some children who do not engage with primary care, but also because CPRD participating practices may perform better. We found illness consultation rates in the community and rates of unplanned admission in children were comparable to recent studies [7, 14, 26].

### Implications for research and policy

Our findings support a body of evidence in favour of UHC of primary care to add to efforts to reduce the burden of disease in the population and alleviate strain on hospital services from unplanned admissions [3, 8, 27]. The high level of infant immunisation coverage across all four nations in the UK in recent years is a success story that other nations have yet to achieve [28]. UHC is an important means of improving health equity, especially essential in lower-income settings. However, even in high-income countries, we found a significantly higher risk for hospital admission for children living below the poorest quintile, demonstrating additional room for improvement in countries with UCH and that service provision alone is not enough. Our findings suggest preventive primary care may help to reduce unplanned admissions, particularly in infants who have the highest contact rates with primary care. Nevertheless, health services beyond infancy tend to be responsive rather than anticipatory, which may disadvantage children with chronic illness.

The UK health system currently lacks a clear framework for the health and wellbeing of children beyond infant immunisation. The healthcare needs of children differ in important ways from those of adults; with greater dependency on parents, education remains an important component of families’ ability to care. In the United States, potentially preventable admissions among children have been decreasing [29]. Future work could explore cost effectiveness of new models of care such as GP-led urgent care centres and impact of certain core features of primary care such as access, structured preventive care, continuity of care and case management of children with chronic conditions [30]. We need to better understand how primary care can serve the needs of children with long-term or complex conditions.

### Limitations

Data quality and extensive coverage of the CPRD database [11] reduces the possibility that our findings are due to chance but, as with any observational cohort study, there are several important biases relating to coding accuracy, completeness and losses to follow-up [31]. A main source of bias in our findings likely arises due to losses to follow-up across the period; however, these were minimised by the way our cohort was constructed such that children contributed time in the cohort according to their age band. We excluded children registered at practices without HES and mother and baby linkage. We found children living in deprived areas were more likely to deregister than children from affluent groups; therefore, our findings are not likely to be generalisable to these children, who may represent a more mobile population and may require a different policy response [32]. However, our sensitivity analysis suggests findings are similar for these families although effect sizes of associations are diluted by their loss to follow-up over the period. We did not obtain health records for the mother, restricting our ability to investigate further health maternal factors or those arising from birth.

Our analysis was highly powered and all reported associations in the full cohort had a significance level greater than 99%. However, in the ACS cohorts, numbers were much smaller, for example, only 268 (0.2%) children aged 5–9 years had a diagnosis of diabetes. As a result, we lacked power to identify association between preventive care and unplanned admissions in children with diabetes.

Our measure of incomplete vaccination may be an underestimate. Determining whether children in birth cohorts, born over several birth years, have received all their primary immunisations is complex given the relatively frequent changes to the detail of the nationally recommended immunisation schedule. In some years, catch up immunisations took place, increasing the basic number [33, 34].

There is residual confounding in our study comparing children with delayed or incomplete vaccination with those who engage with the immunisation programme. These groups are likely to differ in important ways relating to their underlying health, health seeking behaviour and health beliefs, which could also impact on their risk for admission to hospital.

Although most routine preventive care occurs in the community, our data sources would not capture preventive care such as developmental check-ups and vaccinations received in specialist settings. This may be the case in a small minority of children, for example, those with preterm birth or congenital conditions.

Our methodologies, calculating within practice variation and marginal means, reduce the impact of practice level confounding and our sensitivity analyses suggest that practice level confounding may exist in infants but has little effect in older children. Differentiating between health status and illness severity is challenging given a lack of reliable indicators in routine data. In a UHC system, consultations with GPs are not a good proxy for poor health status since preventive advice or disease management for ACS chronic conditions can be given during a consultation; this is highlighted by the fact that illness consultations modify the effect of having an ACS chronic condition on unplanned hospital admissions [35]. Hence, this is among a number of sources of residual confounding that we were unable to account for.

## Conclusions

A high uptake of preventive primary care, including vaccination and development checks, is associated with fewer unplanned and ACS admissions from birth and through childhood. However, the clustering of poor health, a lack of preventive care uptake, and social deprivation puts some children with comorbid conditions at very high risk of admission. Countries with poor immunisation coverage and preventive primary care could significantly reduce the health burden from hospital admission in children, especially those with ACS conditions, through strengthening UHC and primary care.

## Additional files

Additional file 1: **Table S1.** Read codes identifying preventive care consultations and children with a coded diagnosis of an ambulatory care sensitive condition. **Table S2.** Ambulatory care sensitive admission ICD-10 codes. **Table S3.** International Classification of Disease version 10 (ICD-10) diagnoses for prematurity/low birth weight. **Table S4.** ICD-10 diagnoses for congenital disease. **Table S5.** Covariates and outcomes in children with full versus censored follow-up. **Table S6.** Adjusted hazard ratios for unplanned admission stratified by full versus censored follow-up in infants. **Table S7.** Association of preventive primary care, comorbidity and social factors on risk of unplanned hospital admission in using a random intercept model clustering by GP practice. (DOCX 107 kb)

Additional file 2: A supplementary file detailing the methodology used for creating the birth cohort; the preventive care consultations and illness consultations. (DOCX 24 kb)

## Abbreviations

ACS: ambulatory care sensitive
CPRD: Clinical Practice Research Datalink
GP: general practitioner
HES: Hospital Episode Statistics
ICD-10: International Classification of Diseases version 10
NHS: National Health Service
UHC: universal health care
UK: United Kingdom
