# Supplementary material for: Impact of preventive primary care on children’s unplanned hospital admissions: a population-based birth cohort study of UK children 2000–2013
Source: BMC Med. 2018 Sep 17;16:151. doi: 10.1186/s12916-018-1142-3 (PMC6139908; doi:10.1186/s12916-018-1142-3)
Supplement: Supplementary file 1 — Table S1. Read codes identifying preventive care consultations and children with a coded diagnosis of an ambulatory care sensitive condition. Table S2. Ambulatory care sensitive admission ICD-10 codes. Table S3. International Classification of Disease version 10 (ICD-10) diagnoses for prematurity/low birth weight. Table S4. ICD-10 diagnoses for congenital disease. Table S5. Covariates and outcomes in children with full versus censored follow-up. Table S6. Adjusted hazard ratios for unplanned admission stratified by full versus censored follow-up in infants. Table S7. Association of preventive primary care, comorbidity and social factors on risk of unplanned hospital admission in using a random intercept model clustering by GP practice. (DOCX 107 kb) [file 12916_2018_1142_MOESM1_ESM.docx]

**Supplementary Tables**

Table S1: Read Codes identifying preventive care consultations and children with a coded diagnosis of an ambulatory care sensitive condition

| **Description** | **V2 Read Codes** |
| --- | --- |
| **Preventive care** |  |
| Child development checks | 64… |
| Routine child health check | ZV202 |
| Vaccinations | All vaccinations (from CPRD immunization files) |
| **Diagnosis Codes** |  |
| Asthma | H33, (Excluding H333)  H3120, 173A |
| Diabetes | C10.., C109J, C109K, C10C., C10D., C10E.%,  C10F.% (excluding C10F8), C10G.%, C10H.%, C10M.%, C10N.%, PKyP., C10P.% |
| Epilepsy | F25..% (excluding F2501, F2504, F2511, F2516, F256.%, F258. – F25A., F25y4, F25G., F25H.)  F1321 SC200 |

Table S2: Ambulatory care sensitive admission ICD-10 codes

| **Description** | **ICD-10 codes** |
| --- | --- |
| **Chronic conditions** |  |
| Asthma | J45, J46 |
| Diabetes | E10-E14 |
| Epilepsy | G40, G41 |
| **Acute infections** |  |
| Lower respiratory tract infections | J10.0, J11.0, J11.1, J12-J16, J18.0, J18.1, J18.9, J21 |
| Upper respiratory tract infections | H66, H67, J02, J03, J04.0, J06, J31.2 |
| Dehydration and gastroenteritis | E86, K52.2, K52.8, K52.9, A02.0, A04, A07.2, A08.0, A08.1, A08.3, A08.4, A08.5, A09 |
| Urinary tract infections | N10-N12, N13.6, N15.9, N30.0, N30.8, N30.9, N39.0 |
| **Other** |  |
| Vaccine-preventable diseases | A35-A37, A80, B05, B06, B16.1, B16.9, B18.0, B18.1, B26, G00.0, M01.4 |

Table S3: International Classification of Disease version 10 (ICD-10) diagnoses for prematurity/low birth weight

| ICD-10 code | ICD10 detail | Diagnosis category |
| --- | --- | --- |
| P05 | Slow fetal growth and fetal malnutrition | Short gestation; low birth weight; and fetal growth retardation |
| P05.0 | Light for gestational age | Short gestation; low birth weight; and fetal growth retardation |
| P05.1 | Small for gestational age | Short gestation; low birth weight; and fetal growth retardation |
| P05.2 | Newborn affected by fetal (intrauterine) malnutrition not light or small for gestational age | Short gestation; low birth weight; and fetal growth retardation |
| P05.9 | Newborn affected by slow intrauterine growth, unspecified | Short gestation; low birth weight; and fetal growth retardation |
| P07 | Disorders related to short gestation and low birth weight, not elsewhere classified | Short gestation; low birth weight; and fetal growth retardation |
| P07.0 | Extremely low birth weight | Short gestation; low birth weight; and fetal growth retardation |
| P07.1 | Other low birth weight | Short gestation; low birth weight; and fetal growth retardation |
| P07.2 | Extreme immaturity | Short gestation; low birth weight; and fetal growth retardation |
| P07.3 | Other preterm infants | Short gestation; low birth weight; and fetal growth retardation |

Table S4: International Classification of Disease version 10 (ICD-10) diagnoses for congenital disease

| ICD-10 code | ICD10 detail | Diagnosis category |
| --- | --- | --- |
| E84 | Cystic fibrosis | Cystic fibrosis |
| E84.0 | Cystic fibrosis with pulmonary manifestations | Cystic fibrosis |
| E84.1 | Cystic fibrosis with intestinal manifestations | Cystic fibrosis |
| E84.8 | Cystic fibrosis with other manifestations | Cystic fibrosis |
| E84.9 | Cystic fibrosis, unspecified | Cystic fibrosis |
| D80 | Immunodeficiency with predominantly antibody defects | Immunity disorders |
| D80.0 | Hereditary hypogammaglobulinemia | Immunity disorders |
| D80.1 | Nonfamilial hypogammaglobulinemia | Immunity disorders |
| D80.2 | Selective deficiency of immunoglobulin A [IgA] | Immunity disorders |
| D80.3 | Selective deficiency of immunoglobulin G [IgG] subclasses | Immunity disorders |
| D80.4 | Selective deficiency of immunoglobulin M [IgM] | Immunity disorders |
| D80.5 | Immunodeficiency with increased immunoglobulin M [IgM] | Immunity disorders |
| D80.6 | Antibody deficiency with near-normal immunoglobulins or with hyperimmunoglobulinemia | Immunity disorders |
| D80.7 | Transient hypogammaglobulinemia of infancy | Immunity disorders |
| D80.8 | Other immunodeficiencies with predominantly antibody defects | Immunity disorders |
| D80.9 | Immunodeficiency with predominantly antibody defects, unspecified | Immunity disorders |
| D81 | Combined immunodeficiencies | Immunity disorders |
| D81.0 | Severe combined immunodeficiency [SCID] with reticular dysgenesis | Immunity disorders |
| D81.1 | Severe combined immunodeficiency [SCID] with low T- and B-cell numbers | Immunity disorders |
| D81.2 | Severe combined immunodeficiency [SCID] with low or normal B-cell numbers | Immunity disorders |
| D81.3 | Adenosine deaminase [ADA] deficiency | Immunity disorders |
| D81.4 | Nezelofs syndrome | Immunity disorders |
| D81.5 | Purine nucleoside phosphorylase [PNP] deficiency | Immunity disorders |
| D81.6 | Major histocompatibility complex class I deficiency | Immunity disorders |
| D81.7 | Major histocompatibility complex class II deficiency | Immunity disorders |
| D81.8 | Other combined immunodeficiencies | Immunity disorders |
| D81.9 | Combined immunodeficiency, unspecified | Immunity disorders |
| D82 | Immunodeficiency associated with other major defects | Immunity disorders |
| D82.0 | Wiskott-Aldrich syndrome | Immunity disorders |
| D82.1 | Di Georges syndrome | Immunity disorders |
| D82.2 | Immunodeficiency with short-limbed stature | Immunity disorders |
| D82.3 | Immunodeficiency following hereditary defective response to Epstein-Barr virus | Immunity disorders |
| D82.4 | Hyperimmunoglobulin E [IgE] syndrome | Immunity disorders |
| D82.8 | Immunodeficiency associated with other specified major defects | Immunity disorders |
| D82.9 | Immunodeficiency associated with major defect, unspecified | Immunity disorders |
| D83 | Common variable immunodeficiency | Immunity disorders |
| D83.0 | Common variable immunodeficiency with predominant abnormalities of B-cell numbers and function | Immunity disorders |
| D83.1 | Common variable immunodeficiency with predominant immunoregulatory T-cell disorders | Immunity disorders |
| D83.2 | Common variable immunodeficiency with autoantibodies to B- or T-cells | Immunity disorders |
| D83.8 | Other common variable immunodeficiencies | Immunity disorders |
| D83.9 | Common variable immunodeficiency, unspecified | Immunity disorders |
| D84 | Other immunodeficiencies | Immunity disorders |
| D84.0 | Lymphocyte function antigen-1 [LFA-1] defect | Immunity disorders |
| D84.1 | Defects in the complement system | Immunity disorders |
| D84.8 | Other specified immunodeficiencies | Immunity disorders |
| D84.9 | Immunodeficiency, unspecified | Immunity disorders |
| D89 | Other disorders involving the immune mechanism, not elsewhere classified | Immunity disorders |
| D89.0 | Polyclonal hypergammaglobulinemia | Immunity disorders |
| D89.1 | Cryoglobulinemia | Immunity disorders |
| D89.2 | Hypergammaglobulinemia, unspecified | Immunity disorders |
| D89.8 | Other specified disorders involving the immune mechanism, not elsewhere classified | Immunity disorders |
| D89.9 | Disorder involving the immune mechanism, unspecified | Immunity disorders |
| P29.3 | Persistent fetal circulation | Cardiac and circulatory congenital anomalies |
| Q20 | Congenital malformations of cardiac chambers and connections | Cardiac and circulatory congenital anomalies |
| Q20.0 | Common arterial trunk | Cardiac and circulatory congenital anomalies |
| Q20.1 | Double outlet right ventricle | Cardiac and circulatory congenital anomalies |
| Q20.2 | Double outlet left ventricle | Cardiac and circulatory congenital anomalies |
| Q20.3 | Discordant ventriculoarterial connection | Cardiac and circulatory congenital anomalies |
| Q20.4 | Double inlet ventricle | Cardiac and circulatory congenital anomalies |
| Q20.5 | Discordant atrioventricular connection | Cardiac and circulatory congenital anomalies |
| Q20.6 | Isomerism of atrial appendages | Cardiac and circulatory congenital anomalies |
| Q20.8 | Other congenital malformations of cardiac chambers and connections | Cardiac and circulatory congenital anomalies |
| Q20.9 | Congenital malformation of cardiac chambers and connections, unspecified | Cardiac and circulatory congenital anomalies |
| Q21 | Congenital malformations of cardiac septa | Cardiac and circulatory congenital anomalies |
| Q21.0 | Ventricular septal defect | Cardiac and circulatory congenital anomalies |
| Q21.1 | Atrial septal defect | Cardiac and circulatory congenital anomalies |
| Q21.2 | Atrioventricular septal defect | Cardiac and circulatory congenital anomalies |
| Q21.3 | Tetralogy of Fallot | Cardiac and circulatory congenital anomalies |
| Q21.4 | Aortopulmonary septal defect | Cardiac and circulatory congenital anomalies |
| Q21.8 | Other congenital malformations of cardiac septa | Cardiac and circulatory congenital anomalies |
| Q21.9 | Congenital malformation of cardiac septum, unspecified | Cardiac and circulatory congenital anomalies |
| Q22 | Congenital malformations of pulmonary and tricuspid valves | Cardiac and circulatory congenital anomalies |
| Q22.0 | Pulmonary valve atresia | Cardiac and circulatory congenital anomalies |
| Q22.1 | Congenital pulmonary valve stenosis | Cardiac and circulatory congenital anomalies |
| Q22.2 | Congenital pulmonary valve insufficiency | Cardiac and circulatory congenital anomalies |
| Q22.3 | Other congenital malformations of pulmonary valve | Cardiac and circulatory congenital anomalies |
| Q22.4 | Congenital tricuspid stenosis | Cardiac and circulatory congenital anomalies |
| Q22.5 | Ebsteins anomaly | Cardiac and circulatory congenital anomalies |
| Q22.6 | Hypoplastic right heart syndrome | Cardiac and circulatory congenital anomalies |
| Q22.8 | Other congenital malformations of tricuspid valve | Cardiac and circulatory congenital anomalies |
| Q22.9 | Congenital malformation of tricuspid valve, unspecified | Cardiac and circulatory congenital anomalies |
| Q23 | Congenital malformations of aortic and mitral valves | Cardiac and circulatory congenital anomalies |
| Q23.0 | Congenital stenosis of aortic valve | Cardiac and circulatory congenital anomalies |
| Q23.1 | Congenital insufficiency of aortic valve | Cardiac and circulatory congenital anomalies |
| Q23.2 | Congenital mitral stenosis | Cardiac and circulatory congenital anomalies |
| Q23.3 | Congenital mitral insufficiency | Cardiac and circulatory congenital anomalies |
| Q23.4 | Hypoplastic left heart syndrome | Cardiac and circulatory congenital anomalies |
| Q23.8 | Other congenital malformations of aortic and mitral valves | Cardiac and circulatory congenital anomalies |
| Q23.9 | Congenital malformation of aortic and mitral valves, unspecified | Cardiac and circulatory congenital anomalies |
| Q24 | Other congenital malformations of heart | Cardiac and circulatory congenital anomalies |
| Q24.0 | Dextrocardia | Cardiac and circulatory congenital anomalies |
| Q24.1 | Levocardia | Cardiac and circulatory congenital anomalies |
| Q24.2 | Cor triatriatum | Cardiac and circulatory congenital anomalies |
| Q24.3 | Pulmonary infundibular stenosis | Cardiac and circulatory congenital anomalies |
| Q24.4 | Congenital subaortic stenosis | Cardiac and circulatory congenital anomalies |
| Q24.5 | Malformation of coronary vessels | Cardiac and circulatory congenital anomalies |
| Q24.6 | Congenital heart block | Cardiac and circulatory congenital anomalies |
| Q24.8 | Other specified congenital malformations of heart | Cardiac and circulatory congenital anomalies |
| Q24.9 | Congenital malformation of heart, unspecified | Cardiac and circulatory congenital anomalies |
| Q25 | Congenital malformations of great arteries | Cardiac and circulatory congenital anomalies |
| Q25.0 | Patent ductus arteriosus | Cardiac and circulatory congenital anomalies |
| Q25.1 | Coarctation of aorta | Cardiac and circulatory congenital anomalies |
| Q25.2 | Atresia of aorta | Cardiac and circulatory congenital anomalies |
| Q25.3 | Supravalvular aortic stenosis | Cardiac and circulatory congenital anomalies |
| Q25.4 | Other congenital malformations of aorta | Cardiac and circulatory congenital anomalies |
| Q25.5 | Atresia of pulmonary artery | Cardiac and circulatory congenital anomalies |
| Q25.6 | Stenosis of pulmonary artery | Cardiac and circulatory congenital anomalies |
| Q25.7 | Other congenital malformations of pulmonary artery | Cardiac and circulatory congenital anomalies |
| Q25.8 | Other congenital malformations of other great arteries | Cardiac and circulatory congenital anomalies |
| Q25.9 | Congenital malformation of great arteries, unspecified | Cardiac and circulatory congenital anomalies |
| Q26 | Congenital malformations of great veins | Cardiac and circulatory congenital anomalies |
| Q26.0 | Congenital stenosis of vena cava | Cardiac and circulatory congenital anomalies |
| Q26.1 | Persistent left superior vena cava | Cardiac and circulatory congenital anomalies |
| Q26.2 | Total anomalous pulmonary venous connection | Cardiac and circulatory congenital anomalies |
| Q26.3 | Partial anomalous pulmonary venous connection | Cardiac and circulatory congenital anomalies |
| Q26.4 | Anomalous pulmonary venous connection, unspecified | Cardiac and circulatory congenital anomalies |
| Q26.5 | Anomalous portal venous connection | Cardiac and circulatory congenital anomalies |
| Q26.6 | Portal vein-hepatic artery fistula | Cardiac and circulatory congenital anomalies |
| Q26.8 | Other congenital malformations of great veins | Cardiac and circulatory congenital anomalies |
| Q26.9 | Congenital malformation of great vein, unspecified | Cardiac and circulatory congenital anomalies |
| Q27 | Other congenital malformations of peripheral vascular system | Cardiac and circulatory congenital anomalies |
| Q27.0 | Congenital absence and hypoplasia of umbilical artery | Cardiac and circulatory congenital anomalies |
| Q27.1 | Congenital renal artery stenosis | Cardiac and circulatory congenital anomalies |
| Q27.2 | Other congenital malformations of renal artery | Cardiac and circulatory congenital anomalies |
| Q27.3 | Peripheral arteriovenous malformation | Cardiac and circulatory congenital anomalies |
| Q27.4 | Congenital phlebectasia | Cardiac and circulatory congenital anomalies |
| Q27.8 | Other specified congenital malformations of peripheral vascular system | Cardiac and circulatory congenital anomalies |
| Q27.9 | Congenital malformation of peripheral vascular system, unspecified | Cardiac and circulatory congenital anomalies |
| Q28 | Other congenital malformations of circulatory system | Cardiac and circulatory congenital anomalies |
| Q28.0 | Arteriovenous malformation of precerebral vessels | Cardiac and circulatory congenital anomalies |
| Q28.1 | Other malformations of precerebral vessels | Cardiac and circulatory congenital anomalies |
| Q28.2 | Arteriovenous malformation of cerebral vessels | Cardiac and circulatory congenital anomalies |
| Q28.3 | Other malformations of cerebral vessels | Cardiac and circulatory congenital anomalies |
| Q28.8 | Other specified congenital malformations of circulatory system | Cardiac and circulatory congenital anomalies |
| Q28.9 | Congenital malformation of circulatory system, unspecified | Cardiac and circulatory congenital anomalies |
| Q00 | Anencephaly and similar malformations | Nervous system congenital anomalies |
| Q00.0 | Anencephaly | Nervous system congenital anomalies |
| Q00.1 | Craniorachischisis | Nervous system congenital anomalies |
| Q00.2 | Iniencephaly | Nervous system congenital anomalies |
| Q01 | Encephalocele | Nervous system congenital anomalies |
| Q01.0 | Frontal encephalocele | Nervous system congenital anomalies |
| Q01.1 | Nasofrontal encephalocele | Nervous system congenital anomalies |
| Q01.2 | Occipital encephalocele | Nervous system congenital anomalies |
| Q01.8 | Encephalocele of other sites | Nervous system congenital anomalies |
| Q01.9 | Encephalocele, unspecified | Nervous system congenital anomalies |
| Q02 | Microcephaly | Nervous system congenital anomalies |
| Q03 | Congenital hydrocephalus | Nervous system congenital anomalies |
| Q03.0 | Malformations of aqueduct of Sylvius | Nervous system congenital anomalies |
| Q03.1 | Atresia of foramina of Magendie and Luschka | Nervous system congenital anomalies |
| Q03.8 | Other congenital hydrocephalus | Nervous system congenital anomalies |
| Q03.9 | Congenital hydrocephalus, unspecified | Nervous system congenital anomalies |
| Q04 | Other congenital malformations of brain | Nervous system congenital anomalies |
| Q04.0 | Congenital malformations of corpus callosum | Nervous system congenital anomalies |
| Q04.1 | Arhinencephaly | Nervous system congenital anomalies |
| Q04.2 | Holoprosencephaly | Nervous system congenital anomalies |
| Q04.3 | Other reduction deformities of brain | Nervous system congenital anomalies |
| Q04.4 | Septo-optic dysplasia of brain | Nervous system congenital anomalies |
| Q04.5 | Megalencephaly | Nervous system congenital anomalies |
| Q04.6 | Congenital cerebral cysts | Nervous system congenital anomalies |
| Q04.8 | Other specified congenital malformations of brain | Nervous system congenital anomalies |
| Q04.9 | Congenital malformation of brain, unspecified | Nervous system congenital anomalies |
| Q05 | Spina bifida | Nervous system congenital anomalies |
| Q05.0 | Cervical spina bifida with hydrocephalus | Nervous system congenital anomalies |
| Q05.1 | Thoracic spina bifida with hydrocephalus | Nervous system congenital anomalies |
| Q05.2 | Lumbar spina bifida with hydrocephalus | Nervous system congenital anomalies |
| Q05.3 | Sacral spina bifida with hydrocephalus | Nervous system congenital anomalies |
| Q05.4 | Unspecified spina bifida with hydrocephalus | Nervous system congenital anomalies |
| Q05.5 | Cervical spina bifida without hydrocephalus | Nervous system congenital anomalies |
| Q05.6 | Thoracic spina bifida without hydrocephalus | Nervous system congenital anomalies |
| Q05.7 | Lumbar spina bifida without hydrocephalus | Nervous system congenital anomalies |
| Q05.8 | Sacral spina bifida without hydrocephalus | Nervous system congenital anomalies |
| Q05.9 | Spina bifida, unspecified | Nervous system congenital anomalies |
| Q06 | Other congenital malformations of spinal cord | Nervous system congenital anomalies |
| Q06.0 | Amyelia | Nervous system congenital anomalies |
| Q06.1 | Hypoplasia and dysplasia of spinal cord | Nervous system congenital anomalies |
| Q06.2 | Diastematomyelia | Nervous system congenital anomalies |
| Q06.3 | Other congenital cauda equina malformations | Nervous system congenital anomalies |
| Q06.4 | Hydromyelia | Nervous system congenital anomalies |
| Q06.8 | Other specified congenital malformations of spinal cord | Nervous system congenital anomalies |
| Q06.9 | Congenital malformation of spinal cord, unspecified | Nervous system congenital anomalies |
| Q07 | Other congenital malformations of nervous system | Nervous system congenital anomalies |
| Q07.0 | Arnold-Chiari syndrome | Nervous system congenital anomalies |
| Q07.8 | Other specified congenital malformations of nervous system | Nervous system congenital anomalies |
| Q07.9 | Congenital malformation of nervous system, unspecified | Nervous system congenital anomalies |
| Q10 | Congenital malformations of eyelid, lacrimal apparatus and orbit | Other congenital anomalies |
| Q10.0 | Congenital ptosis | Other congenital anomalies |
| Q10.1 | Congenital ectropion | Other congenital anomalies |
| Q10.2 | Congenital entropion | Other congenital anomalies |
| Q10.3 | Other congenital malformations of eyelid | Other congenital anomalies |
| Q10.4 | Absence and agenesis of lacrimal apparatus | Other congenital anomalies |
| Q10.5 | Congenital stenosis and stricture of lacrimal duct | Other congenital anomalies |
| Q10.6 | Other congenital malformations of lacrimal apparatus | Other congenital anomalies |
| Q10.7 | Congenital malformation of orbit | Other congenital anomalies |
| Q11 | Anophthalmos, microphthalmos and macrophthalmos | Other congenital anomalies |
| Q11.0 | Cystic eyeball | Other congenital anomalies |
| Q11.1 | Other anophthalmos | Other congenital anomalies |
| Q11.2 | Microphthalmos | Other congenital anomalies |
| Q11.3 | Macrophthalmos | Other congenital anomalies |
| Q12 | Congenital lens malformations | Other congenital anomalies |
| Q12.0 | Congenital cataract | Other congenital anomalies |
| Q12.1 | Congenital displaced lens | Other congenital anomalies |
| Q12.2 | Coloboma of lens | Other congenital anomalies |
| Q12.3 | Congenital aphakia | Other congenital anomalies |
| Q12.4 | Spherophakia | Other congenital anomalies |
| Q12.8 | Other congenital lens malformations | Other congenital anomalies |
| Q12.9 | Congenital lens malformation, unspecified | Other congenital anomalies |
| Q13 | Congenital malformations of anterior segment of eye | Other congenital anomalies |
| Q13.0 | Coloboma of iris | Other congenital anomalies |
| Q13.1 | Absence of iris | Other congenital anomalies |
| Q13.2 | Other congenital malformations of iris | Other congenital anomalies |
| Q13.3 | Congenital corneal opacity | Other congenital anomalies |
| Q13.4 | Other congenital corneal malformations | Other congenital anomalies |
| Q13.5 | Blue sclera | Other congenital anomalies |
| Q13.8 | Other congenital malformations of anterior segment of eye | Other congenital anomalies |
| Q13.9 | Congenital malformation of anterior segment of eye, unspecified | Other congenital anomalies |
| Q14 | Congenital malformations of posterior segment of eye | Other congenital anomalies |
| Q14.0 | Congenital malformation of vitreous humor | Other congenital anomalies |
| Q14.1 | Congenital malformation of retina | Other congenital anomalies |
| Q14.2 | Congenital malformation of optic disc | Other congenital anomalies |
| Q14.3 | Congenital malformation of choroid | Other congenital anomalies |
| Q14.8 | Other congenital malformations of posterior segment of eye | Other congenital anomalies |
| Q14.9 | Congenital malformation of posterior segment of eye, unspecified | Other congenital anomalies |
| Q15 | Other congenital malformations of eye | Other congenital anomalies |
| Q15.0 | Congenital glaucoma | Other congenital anomalies |
| Q15.8 | Other specified congenital malformations of eye | Other congenital anomalies |
| Q15.9 | Congenital malformation of eye, unspecified | Other congenital anomalies |
| Q16 | Congenital malformations of ear causing impairment of hearing | Other congenital anomalies |
| Q16.0 | Congenital absence of (ear) auricle | Other congenital anomalies |
| Q16.1 | Congenital absence, atresia and stricture of auditory canal (external) | Other congenital anomalies |
| Q16.2 | Absence of eustachian tube | Other congenital anomalies |
| Q16.3 | Congenital malformation of ear ossicles | Other congenital anomalies |
| Q16.4 | Other congenital malformations of middle ear | Other congenital anomalies |
| Q16.5 | Congenital malformation of inner ear | Other congenital anomalies |
| Q16.9 | Congenital malformation of ear causing impairment of hearing, unspecified | Other congenital anomalies |
| Q17 | Other congenital malformations of ear | Other congenital anomalies |
| Q17.0 | Accessory auricle | Other congenital anomalies |
| Q17.1 | Macrotia | Other congenital anomalies |
| Q17.2 | Microtia | Other congenital anomalies |
| Q17.3 | Other misshapen ear | Other congenital anomalies |
| Q17.4 | Misplaced ear | Other congenital anomalies |
| Q17.5 | Prominent ear | Other congenital anomalies |
| Q17.8 | Other specified congenital malformations of ear | Other congenital anomalies |
| Q17.9 | Congenital malformation of ear, unspecified | Other congenital anomalies |
| Q18 | Other congenital malformations of face and neck | Other congenital anomalies |
| Q18.0 | Sinus, fistula and cyst of branchial cleft | Other congenital anomalies |
| Q18.1 | Preauricular sinus and cyst | Other congenital anomalies |
| Q18.2 | Other branchial cleft malformations | Other congenital anomalies |
| Q18.3 | Webbing of neck | Other congenital anomalies |
| Q18.4 | Macrostomia | Other congenital anomalies |
| Q18.5 | Microstomia | Other congenital anomalies |
| Q18.6 | Macrocheilia | Other congenital anomalies |
| Q18.7 | Microcheilia | Other congenital anomalies |
| Q18.8 | Other specified congenital malformations of face and neck | Other congenital anomalies |
| Q18.9 | Congenital malformation of face and neck, unspecified | Other congenital anomalies |
| Q30 | Congenital malformations of nose | Other congenital anomalies |
| Q30.0 | Choanal atresia | Other congenital anomalies |
| Q30.1 | Agenesis and underdevelopment of nose | Other congenital anomalies |
| Q30.2 | Fissured, notched and cleft nose | Other congenital anomalies |
| Q30.3 | Congenital perforated nasal septum | Other congenital anomalies |
| Q30.8 | Other congenital malformations of nose | Other congenital anomalies |
| Q30.9 | Congenital malformation of nose, unspecified | Other congenital anomalies |
| Q31 | Congenital malformations of larynx | Other congenital anomalies |
| Q31.0 | Web of larynx | Other congenital anomalies |
| Q31.1 | Congenital subglottic stenosis | Other congenital anomalies |
| Q31.2 | Laryngeal hypoplasia | Other congenital anomalies |
| Q31.3 | Laryngocele | Other congenital anomalies |
| Q31.4 | Congenital laryngeal stridor | Other congenital anomalies |
| Q31.5 | Congenital laryngomalacia | Other congenital anomalies |
| Q31.8 | Other congenital malformations of larynx | Other congenital anomalies |
| Q31.9 | Congenital malformation of larynx, unspecified | Other congenital anomalies |
| Q32 | Congenital malformations of trachea and bronchus | Other congenital anomalies |
| Q32.0 | Congenital tracheomalacia | Other congenital anomalies |
| Q32.1 | Other congenital malformations of trachea | Other congenital anomalies |
| Q32.2 | Congenital bronchomalacia | Other congenital anomalies |
| Q32.3 | Congenital stenosis of bronchus | Other congenital anomalies |
| Q32.4 | Other congenital malformations of bronchus | Other congenital anomalies |
| Q33 | Congenital malformations of lung | Other congenital anomalies |
| Q33.0 | Congenital cystic lung | Other congenital anomalies |
| Q33.1 | Accessory lobe of lung | Other congenital anomalies |
| Q33.2 | Sequestration of lung | Other congenital anomalies |
| Q33.3 | Agenesis of lung | Other congenital anomalies |
| Q33.4 | Congenital bronchiectasis | Other congenital anomalies |
| Q33.5 | Ectopic tissue in lung | Other congenital anomalies |
| Q33.6 | Congenital hypoplasia and dysplasia of lung | Other congenital anomalies |
| Q33.8 | Other congenital malformations of lung | Other congenital anomalies |
| Q33.9 | Congenital malformation of lung, unspecified | Other congenital anomalies |
| Q34 | Other congenital malformations of respiratory system | Other congenital anomalies |
| Q34.0 | Anomaly of pleura | Other congenital anomalies |
| Q34.1 | Congenital cyst of mediastinum | Other congenital anomalies |
| Q34.8 | Other specified congenital malformations of respiratory system | Other congenital anomalies |
| Q34.9 | Congenital malformation of respiratory system, unspecified | Other congenital anomalies |
| Q35 | Cleft palate | Other congenital anomalies |
| Q35.0 | Cleft hard palate, bilater | Other congenital anomalies |
| Q35.1 | Cleft hard palate | Other congenital anomalies |
| Q35.2 | Cleft soft palate, bilater | Other congenital anomalies |
| Q35.3 | Cleft soft palate | Other congenital anomalies |
| Q35.4 | Cleft hard palate with cle | Other congenital anomalies |
| Q35.5 | Cleft hard palate with cleft soft palate | Other congenital anomalies |
| Q35.6 | Cleft palate, medial | Other congenital anomalies |
| Q35.7 | Cleft uvula | Other congenital anomalies |
| Q35.8 | Cleft palate, unspecified, | Other congenital anomalies |
| Q35.9 | Cleft palate, unspecified | Other congenital anomalies |
| Q36 | Cleft lip | Other congenital anomalies |
| Q36.0 | Cleft lip, bilateral | Other congenital anomalies |
| Q36.1 | Cleft lip, median | Other congenital anomalies |
| Q36.9 | Cleft lip, unilateral | Other congenital anomalies |
| Q37 | Cleft palate with cleft lip | Other congenital anomalies |
| Q37.0 | Cleft hard palate with bilateral cleft lip | Other congenital anomalies |
| Q37.1 | Cleft hard palate with unilateral cleft lip | Other congenital anomalies |
| Q37.2 | Cleft soft palate with bilateral cleft lip | Other congenital anomalies |
| Q37.3 | Cleft soft palate with unilateral cleft lip | Other congenital anomalies |
| Q37.4 | Cleft hard and soft palate with bilateral cleft lip | Other congenital anomalies |
| Q37.5 | Cleft hard and soft palate with unilateral cleft lip | Other congenital anomalies |
| Q37.8 | Unspecified cleft palate with bilateral cleft lip | Other congenital anomalies |
| Q37.9 | Unspecified cleft palate with unilateral cleft lip | Other congenital anomalies |
| Q65 | Congenital deformities of hip | Other congenital anomalies |
| Q65.0 | Congenital dislocation of hip, unilateral | Other congenital anomalies |
| Q65.1 | Congenital dislocation of hip, bilateral | Other congenital anomalies |
| Q65.2 | Congenital dislocation of hip, unspecified | Other congenital anomalies |
| Q65.3 | Congenital subluxation of hip, unilateral | Other congenital anomalies |
| Q65.4 | Congenital partial dislocation of hip, bilateral | Other congenital anomalies |
| Q65.5 | Congenital partial dislocation of hip, unspecified | Other congenital anomalies |
| Q65.6 | Congenital unstable hip | Other congenital anomalies |
| Q65.8 | Other congenital deformities of hip | Other congenital anomalies |
| Q65.9 | Congenital deformity of hip, unspecified | Other congenital anomalies |
| Q66 | Congenital deformities of feet | Other congenital anomalies |
| Q66.0 | Congenital talipes equinovarus | Other congenital anomalies |
| Q66.1 | Congenital talipes calcaneovarus | Other congenital anomalies |
| Q66.2 | Congenital metatarsus (primus) varus | Other congenital anomalies |
| Q66.3 | Other congenital varus deformities of feet | Other congenital anomalies |
| Q66.4 | Congenital talipes calcaneovalgus | Other congenital anomalies |
| Q66.5 | Congenital pes planus | Other congenital anomalies |
| Q66.6 | Other congenital valgus deformities of feet | Other congenital anomalies |
| Q66.7 | Congenital pes cavus | Other congenital anomalies |
| Q66.8 | Other congenital deformities of feet | Other congenital anomalies |
| Q66.9 | Congenital deformity of feet, unspecified | Other congenital anomalies |
| Q67 | Congenital musculoskeletal deformities of head, face, spine and chest | Other congenital anomalies |
| Q67.0 | Congenital facial asymmetry | Other congenital anomalies |
| Q67.1 | Congenital compression facies | Other congenital anomalies |
| Q67.2 | Dolichocephaly | Other congenital anomalies |
| Q67.3 | Plagiocephaly | Other congenital anomalies |
| Q67.4 | Other congenital deformities of skull, face and jaw | Other congenital anomalies |
| Q67.5 | Congenital deformity of spine | Other congenital anomalies |
| Q67.6 | Pectus excavatum | Other congenital anomalies |
| Q67.7 | Pectus carinatum | Other congenital anomalies |
| Q67.8 | Other congenital deformities of chest | Other congenital anomalies |
| Q68 | Other congenital musculoskeletal deformities | Other congenital anomalies |
| Q68.0 | Congenital deformity of sternocleidomastoid muscle | Other congenital anomalies |
| Q68.1 | Congenital deformity of finger(s) and hand | Other congenital anomalies |
| Q68.2 | Congenital deformity of knee | Other congenital anomalies |
| Q68.3 | Congenital bowing of femur | Other congenital anomalies |
| Q68.4 | Congenital bowing of tibia and fibula | Other congenital anomalies |
| Q68.5 | Congenital bowing of long bones of leg, unspecified | Other congenital anomalies |
| Q68.8 | Other specified congenital musculoskeletal deformities | Other congenital anomalies |
| Q69 | Polydactyly | Other congenital anomalies |
| Q69.0 | Accessory finger(s) | Other congenital anomalies |
| Q69.1 | Accessory thumb(s) | Other congenital anomalies |
| Q69.2 | Accessory toe(s) | Other congenital anomalies |
| Q69.9 | Polydactyly, unspecified | Other congenital anomalies |
| Q70 | Syndactyly | Other congenital anomalies |
| Q70.0 | Fused fingers | Other congenital anomalies |
| Q70.1 | Webbed fingers | Other congenital anomalies |
| Q70.2 | Fused toes | Other congenital anomalies |
| Q70.3 | Webbed toes | Other congenital anomalies |
| Q70.4 | Polysyndactyly | Other congenital anomalies |
| Q70.9 | Syndactyly, unspecified | Other congenital anomalies |
| Q71 | Reduction defects of upper limb | Other congenital anomalies |
| Q71.0 | Congenital complete absence of upper limb(s) | Other congenital anomalies |
| Q71.1 | Congenital absence of upper arm and forearm with hand present | Other congenital anomalies |
| Q71.2 | Congenital absence of both forearm and hand | Other congenital anomalies |
| Q71.3 | Congenital absence of hand and finger(s) | Other congenital anomalies |
| Q71.4 | Longitudinal reduction defect of radius | Other congenital anomalies |
| Q71.5 | Longitudinal reduction defect of ulna | Other congenital anomalies |
| Q71.6 | Lobster-claw hand | Other congenital anomalies |
| Q71.8 | Other reduction defects of upper limb(s) | Other congenital anomalies |
| Q71.9 | Reduction defect of upper limb, unspecified | Other congenital anomalies |
| Q72 | Reduction defects of lower limb | Other congenital anomalies |
| Q72.0 | Congenital complete absence of lower limb(s) | Other congenital anomalies |
| Q72.1 | Congenital absence of thigh and lower leg with foot present | Other congenital anomalies |
| Q72.2 | Congenital absence of both lower leg and foot | Other congenital anomalies |
| Q72.3 | Congenital absence of foot and toe(s) | Other congenital anomalies |
| Q72.4 | Longitudinal reduction defect of femur | Other congenital anomalies |
| Q72.5 | Longitudinal reduction defect of tibia | Other congenital anomalies |
| Q72.6 | Longitudinal reduction defect of fibula | Other congenital anomalies |
| Q72.7 | Split foot | Other congenital anomalies |
| Q72.8 | Other reduction defects of lower limb(s) | Other congenital anomalies |
| Q72.9 | Reduction defect of lower limb, unspecified | Other congenital anomalies |
| Q73 | Reduction defects of unspecified limb | Other congenital anomalies |
| Q73.0 | Congenital absence of unspecified limb(s) | Other congenital anomalies |
| Q73.1 | Phocomelia, unspecified limb(s) | Other congenital anomalies |
| Q73.8 | Other reduction defects of unspecified limb(s) | Other congenital anomalies |
| Q74 | Other congenital malformations of limb(s) | Other congenital anomalies |
| Q74.0 | Other congenital malformations of upper limb(s), including shoulder girdle | Other congenital anomalies |
| Q74.1 | Congenital malformation of knee | Other congenital anomalies |
| Q74.2 | Other congenital malformations of lower limb(s), including pelvic girdle | Other congenital anomalies |
| Q74.3 | Arthrogryposis multiplex congenita | Other congenital anomalies |
| Q74.8 | Other specified congenital malformations of limb(s) | Other congenital anomalies |
| Q74.9 | Unspecified congenital malformation of limb(s) | Other congenital anomalies |
| Q75 | Other congenital malformations of skull and face bones | Other congenital anomalies |
| Q75.0 | Craniosynostosis | Other congenital anomalies |
| Q75.1 | Craniofacial dysostosis | Other congenital anomalies |
| Q75.2 | Hypertelorism | Other congenital anomalies |
| Q75.3 | Macrocephaly | Other congenital anomalies |
| Q75.4 | Mandibulofacial dysostosis | Other congenital anomalies |
| Q75.5 | Oculomandibular dysostosis | Other congenital anomalies |
| Q75.8 | Other specified congenital malformations of skull and face bones | Other congenital anomalies |
| Q75.9 | Congenital malformation of skull and face bones, unspecified | Other congenital anomalies |
| Q76 | Congenital malformations of spine and bony thorax | Other congenital anomalies |
| Q76.0 | Spina bifida occulta | Other congenital anomalies |
| Q76.1 | Klippel-Feil syndrome | Other congenital anomalies |
| Q76.2 | Congenital spondylolisthesis | Other congenital anomalies |
| Q76.3 | Congenital scoliosis due to congenital bony malformation | Other congenital anomalies |
| Q76.4 | Other congenital malformations of spine, not associated with scoliosis | Other congenital anomalies |
| Q76.5 | Cervical rib | Other congenital anomalies |
| Q76.6 | Other congenital malformations of ribs | Other congenital anomalies |
| Q76.7 | Congenital malformation of sternum | Other congenital anomalies |
| Q76.8 | Other congenital malformations of bony thorax | Other congenital anomalies |
| Q76.9 | Congenital malformation of bony thorax, unspecified | Other congenital anomalies |
| Q77 | Osteochondrodysplasia with defects of growth of tubular bones and spine | Other congenital anomalies |
| Q77.0 | Achondrogenesis | Other congenital anomalies |
| Q77.1 | Thanatophoric short stature | Other congenital anomalies |
| Q77.2 | Short rib syndrome | Other congenital anomalies |
| Q77.3 | Chondrodysplasia punctata | Other congenital anomalies |
| Q77.4 | Achondroplasia | Other congenital anomalies |
| Q77.5 | Diastrophic dysplasia | Other congenital anomalies |
| Q77.6 | Chondroectodermal dysplasia | Other congenital anomalies |
| Q77.7 | Spondyloepiphyseal dysplasia | Other congenital anomalies |
| Q77.8 | Other osteochondrodysplasia with defects of growth of tubular bones and spine | Other congenital anomalies |
| Q77.9 | Osteochondrodysplasia with defects of growth of tubular bones and spine, unspecified | Other congenital anomalies |
| Q78 | Other osteochondrodysplasias | Other congenital anomalies |
| Q78.0 | Osteogenesis imperfecta | Other congenital anomalies |
| Q78.1 | Polyostotic fibrous dysplasia | Other congenital anomalies |
| Q78.2 | Osteopetrosis | Other congenital anomalies |
| Q78.3 | Progressive diaphyseal dysplasia | Other congenital anomalies |
| Q78.4 | Enchondromatosis | Other congenital anomalies |
| Q78.5 | Metaphyseal dysplasia | Other congenital anomalies |
| Q78.6 | Multiple congenital exostoses | Other congenital anomalies |
| Q78.8 | Other specified osteochondrodysplasias | Other congenital anomalies |
| Q78.9 | Osteochondrodysplasia, unspecified | Other congenital anomalies |
| Q79 | Congenital malformations of the musculoskeletal system, not elsewhere classified | Other congenital anomalies |
| Q79.0 | Congenital diaphragmatic hernia | Other congenital anomalies |
| Q79.1 | Other congenital malformations of diaphragm | Other congenital anomalies |
| Q79.2 | Exomphalos | Other congenital anomalies |
| Q79.3 | Gastroschisis | Other congenital anomalies |
| Q79.4 | Prune belly syndrome | Other congenital anomalies |
| Q79.5 | Other congenital malformations of abdominal wall | Other congenital anomalies |
| Q79.6 | Ehlers-Danlos syndrome | Other congenital anomalies |
| Q79.8 | Other congenital malformations of musculoskeletal system | Other congenital anomalies |
| Q79.9 | Congenital malformation of musculoskeletal system, unspecified | Other congenital anomalies |
| Q80 | Congenital ichthyosis | Other congenital anomalies |
| Q80.0 | Ichthyosis vulgaris | Other congenital anomalies |
| Q80.1 | X-linked ichthyosis | Other congenital anomalies |
| Q80.2 | Lamellar ichthyosis | Other congenital anomalies |
| Q80.3 | Congenital bullous ichthyosiform erythroderma | Other congenital anomalies |
| Q80.4 | Harlequin fetus | Other congenital anomalies |
| Q80.8 | Other congenital ichthyosis | Other congenital anomalies |
| Q80.9 | Congenital ichthyosis, unspecified | Other congenital anomalies |
| Q81 | Epidermolysis bullosa | Other congenital anomalies |
| Q81.0 | Epidermolysis bullosa simplex | Other congenital anomalies |
| Q81.1 | Epidermolysis bullosa letalis | Other congenital anomalies |
| Q81.2 | Epidermolysis bullosa dystrophica | Other congenital anomalies |
| Q81.8 | Other epidermolysis bullosa | Other congenital anomalies |
| Q81.9 | Epidermolysis bullosa, unspecified | Other congenital anomalies |
| Q82 | Other congenital malformations of skin | Other congenital anomalies |
| Q82.0 | Hereditary lymphedema | Other congenital anomalies |
| Q82.1 | Xeroderma pigmentosum | Other congenital anomalies |
| Q82.2 | Mastocytosis | Other congenital anomalies |
| Q82.3 | Incontinentia pigmenti | Other congenital anomalies |
| Q82.4 | Ectodermal dysplasia (anhidrotic) | Other congenital anomalies |
| Q82.5 | Congenital non-neoplastic nevus | Other congenital anomalies |
| Q82.8 | Other specified congenital malformations of skin | Other congenital anomalies |
| Q82.9 | Congenital malformation of skin, unspecified | Other congenital anomalies |
| Q83 | Congenital malformations of breast | Other congenital anomalies |
| Q83.0 | Congenital absence of breast with absent nipple | Other congenital anomalies |
| Q83.1 | Accessory breast | Other congenital anomalies |
| Q83.2 | Absent nipple | Other congenital anomalies |
| Q83.3 | Accessory nipple | Other congenital anomalies |
| Q83.8 | Other congenital malformations of breast | Other congenital anomalies |
| Q83.9 | Congenital malformation of breast, unspecified | Other congenital anomalies |
| Q84 | Other congenital malformations of integument | Other congenital anomalies |
| Q84.0 | Congenital alopecia | Other congenital anomalies |
| Q84.1 | Congenital morphological disturbances of hair, not elsewhere classified | Other congenital anomalies |
| Q84.2 | Other congenital malformations of hair | Other congenital anomalies |
| Q84.3 | Anonychia | Other congenital anomalies |
| Q84.4 | Congenital leukonychia | Other congenital anomalies |
| Q84.5 | Enlarged and hypertrophic nails | Other congenital anomalies |
| Q84.6 | Other congenital malformations of nails | Other congenital anomalies |
| Q84.8 | Other specified congenital malformations of integument | Other congenital anomalies |
| Q84.9 | Congenital malformation of integument, unspecified | Other congenital anomalies |
| Q85 | Phakomatoses, not elsewhere classified | Other congenital anomalies |
| Q85.0 | Neurofibromatosis (nonmalignant) | Other congenital anomalies |
| Q85.1 | Tuberous sclerosis | Other congenital anomalies |
| Q85.8 | Other phakomatoses, not elsewhere classified | Other congenital anomalies |
| Q85.9 | Phakomatosis, unspecified | Other congenital anomalies |
| Q86 | Congenital malformation syndromes due to known exogenous causes, not elsewhere classified | Other congenital anomalies |
| Q86.0 | Fetal alcohol syndrome (dysmorphic) | Other congenital anomalies |
| Q86.1 | Fetal hydantoin syndrome | Other congenital anomalies |
| Q86.2 | Dysmorphism due to warfarin | Other congenital anomalies |
| Q86.8 | Other congenital malformation syndromes due to known exogenous causes | Other congenital anomalies |
| Q87 | Other specified congenital malformation syndromes affecting multiple systems | Other congenital anomalies |
| Q87.0 | Congenital malformation syndromes predominantly affecting facial appearance | Other congenital anomalies |
| Q87.1 | Congenital malformation syndromes predominantly associated with short stature | Other congenital anomalies |
| Q87.2 | Congenital malformation syndromes predominantly involving limbs | Other congenital anomalies |
| Q87.3 | Congenital malformation syndromes involving early overgrowth | Other congenital anomalies |
| Q87.4 | Marfans syndrome | Other congenital anomalies |
| Q87.5 | Other congenital malformation syndromes with other skeletal changes | Other congenital anomalies |
| Q87.8 | Other specified congenital malformation syndromes, not elsewhere classified | Other congenital anomalies |
| Q89 | Other congenital malformations, not elsewhere classified | Other congenital anomalies |
| Q89.0 | Congenital malformations of spleen | Other congenital anomalies |
| Q89.1 | Congenital malformations of adrenal gland | Other congenital anomalies |
| Q89.2 | Congenital malformations of other endocrine glands | Other congenital anomalies |
| Q89.3 | Situs inversus | Other congenital anomalies |
| Q89.4 | Conjoined twins | Other congenital anomalies |
| Q89.7 | Multiple congenital malformations, not elsewhere classified | Other congenital anomalies |
| Q89.8 | Other specified congenital malformations | Other congenital anomalies |
| Q89.9 | Congenital malformation, unspecified | Other congenital anomalies |
| Q90 | Downs syndrome | Other congenital anomalies |
| Q90.0 | Trisomy 21, nonmosaicism (meiotic nondisjunction) | Other congenital anomalies |
| Q90.1 | Trisomy 21, mosaicism (mitotic nondisjunction) | Other congenital anomalies |
| Q90.2 | Trisomy 21, translocation | Other congenital anomalies |
| Q90.9 | Downs syndrome, unspecified | Other congenital anomalies |
| Q91 | Edwards syndrome and Pataus syndrome | Other congenital anomalies |
| Q91.0 | Trisomy 18, nonmosaicism (meiotic nondisjunction) | Other congenital anomalies |
| Q91.1 | Trisomy 18, mosaicism (mitotic nondisjunction) | Other congenital anomalies |
| Q91.2 | Trisomy 18, translocation | Other congenital anomalies |
| Q91.3 | Trisomy 18, unspecified | Other congenital anomalies |
| Q91.4 | Trisomy 13, nonmosaicism (meiotic nondisjunction) | Other congenital anomalies |
| Q91.5 | Trisomy 13, mosaicism (mitotic nondisjunction) | Other congenital anomalies |
| Q91.6 | Trisomy 13, translocation | Other congenital anomalies |
| Q91.7 | Trisomy 13, unspecified | Other congenital anomalies |
| Q92 | Other trisomies and partial trisomies of the autosomes, not elsewhere classified | Other congenital anomalies |
| Q92.0 | Whole chromosome trisomy, nonmosaicism (meiotic nondisjunction) | Other congenital anomalies |
| Q92.1 | Whole chromosome trisomy, mosaicism (mitotic nondisjunction) | Other congenital anomalies |
| Q92.2 | Partial trisomy | Other congenital anomalies |
| Q92.3 | Minor partial trisomy | Other congenital anomalies |
| Q92.4 | Duplications seen only at prometaphase | Other congenital anomalies |
| Q92.5 | Duplications with other complex rearrangements | Other congenital anomalies |
| Q92.6 | Extra marker chromosomes | Other congenital anomalies |
| Q92.7 | Triploidy and polyploidy | Other congenital anomalies |
| Q92.8 | Other specified trisomies and partial trisomies of autosomes | Other congenital anomalies |
| Q92.9 | Trisomy and partial trisomy of autosomes, unspecified | Other congenital anomalies |
| Q93 | Monosomies and deletions from the autosomes, not elsewhere classified | Other congenital anomalies |
| Q93.0 | Whole chromosome monosomy, nonmosaicism (meiotic nondisjunction) | Other congenital anomalies |
| Q93.1 | Whole chromosome monosomy, mosaicism (mitotic nondisjunction) | Other congenital anomalies |
| Q93.2 | Chromosome replaced with ring, dicentric or isochromosome | Other congenital anomalies |
| Q93.3 | Deletion of short arm of chromosome 4 | Other congenital anomalies |
| Q93.4 | Deletion of short arm of chromosome 5 | Other congenital anomalies |
| Q93.5 | Other deletions of part of a chromosome | Other congenital anomalies |
| Q93.6 | Deletions seen only at prometaphase | Other congenital anomalies |
| Q93.7 | Deletions with other complex rearrangements | Other congenital anomalies |
| Q93.8 | Other deletions from the autosomes | Other congenital anomalies |
| Q93.9 | Deletion from autosomes, unspecified | Other congenital anomalies |
| Q95 | Balanced rearrangements and structural markers, not elsewhere classified | Other congenital anomalies |
| Q95.0 | Balanced translocation and insertion in normal individual | Other congenital anomalies |
| Q95.1 | Chromosome inversion in normal individual | Other congenital anomalies |
| Q95.2 | Balanced autosomal rearrangement in abnormal individual | Other congenital anomalies |
| Q95.3 | Balanced sex/autosomal rearrangement in abnormal individual | Other congenital anomalies |
| Q95.4 | Individuals with marker heterochromatin | Other congenital anomalies |
| Q95.5 | Individual with autosomal fragile site | Other congenital anomalies |
| Q95.8 | Other balanced rearrangements and structural markers | Other congenital anomalies |
| Q95.9 | Balanced rearrangement and structural marker, unspecified | Other congenital anomalies |
| Q96 | Turners syndrome | Other congenital anomalies |
| Q96.0 | Karyotype 45, X | Other congenital anomalies |
| Q96.1 | Karyotype 46, X iso (Xq) | Other congenital anomalies |
| Q96.2 | Karyotype 46, X with abnormal sex chromosome, except iso (Xq) | Other congenital anomalies |
| Q96.3 | Mosaicism, 45, X/46, XX or XY | Other congenital anomalies |
| Q96.4 | Mosaicism, 45, X/other cell line(s) with abnormal sex chromosome | Other congenital anomalies |
| Q96.8 | Other variants of Turners syndrome | Other congenital anomalies |
| Q96.9 | Turners syndrome, unspecified | Other congenital anomalies |
| Q97 | Other sex chromosome abnormalities, female phenotype, not elsewhere classified | Other congenital anomalies |
| Q97.0 | Karyotype 47, XXX | Other congenital anomalies |
| Q97.1 | Female with more than three X chromosomes | Other congenital anomalies |
| Q97.2 | Mosaicism, lines with various numbers of X chromosomes | Other congenital anomalies |
| Q97.3 | Female with 46, XY karyotype | Other congenital anomalies |
| Q97.8 | Other specified sex chromosome abnormalities, female phenotype | Other congenital anomalies |
| Q97.9 | Sex chromosome abnormality, female phenotype, unspecified | Other congenital anomalies |
| Q98 | Other sex chromosome abnormalities, male phenotype, not elsewhere classified | Other congenital anomalies |
| Q98.0 | Klinefelter syndrome karyotype 47, XXY | Other congenital anomalies |
| Q98.1 | Klinefelter syndrome, male with more than two X chromosomes | Other congenital anomalies |
| Q98.2 | Klinefelters syndrome, male with 46,XX karyotype | Other congenital anomalies |
| Q98.3 | Other male with 46, XX karyotype | Other congenital anomalies |
| Q98.4 | Klinefelter syndrome, unspecified | Other congenital anomalies |
| Q98.5 | Karyotype 47, XYY | Other congenital anomalies |
| Q98.6 | Male with structurally abnormal sex chromosome | Other congenital anomalies |
| Q98.7 | Male with sex chromosome mosaicism | Other congenital anomalies |
| Q98.8 | Other specified sex chromosome abnormalities, male phenotype | Other congenital anomalies |
| Q98.9 | Sex chromosome abnormality, male phenotype, unspecified | Other congenital anomalies |
| Q99 | Other chromosome abnormalities, not elsewhere classified | Other congenital anomalies |
| Q99.0 | Chimera 46, XX/46, XY | Other congenital anomalies |
| Q99.1 | 46, XX true hermaphrodite | Other congenital anomalies |
| Q99.2 | Fragile X chromosome | Other congenital anomalies |
| Q99.8 | Other specified chromosome abnormalities | Other congenital anomalies |
| Q99.9 | Chromosomal abnormality, unspecified | Other congenital anomalies |
| R29.4 | Clicking hip | Other congenital anomalies |
| A33 | Tetanus neonatorum | Other perinatal conditions |
| P00 | Fetus and newborn affected by maternal conditions that may be unrelated to present pregnancy | Other perinatal conditions |
| P00.0 | Newborn (suspected to be) affected by maternal hypertensive disorders | Other perinatal conditions |
| P00.1 | Newborn (suspected to be) affected by maternal renal and urinary tract diseases | Other perinatal conditions |
| P00.2 | Newborn (suspected to be) affected by maternal infectious and parasitic diseases | Other perinatal conditions |
| P00.3 | Newborn (suspected to be) affected by other maternal circulatory and respiratory diseases | Other perinatal conditions |
| P00.4 | Newborn (suspected to be) affected by maternal nutritional disorders | Other perinatal conditions |
| P00.5 | Newborn (suspected to be) affected by maternal injury | Other perinatal conditions |
| P00.6 | Newborn (suspected to be) affected by surgical procedure on mother | Other perinatal conditions |
| P00.7 | Newborn (suspected to be) affected by other medical procedures on mother, not elsewhere classified | Other perinatal conditions |
| P00.8 | Fetus and newborn affected by other maternal conditions | Other perinatal conditions |
| P00.9 | Newborn (suspected to be) affected by unspecified maternal condition | Other perinatal conditions |
| P01 | Fetus and newborn affected by maternal complications of pregnancy | Other perinatal conditions |
| P01.0 | Newborn (suspected to be) affected by incompetent cervix | Other perinatal conditions |
| P01.1 | Newborn (suspected to be) affected by premature rupture of membranes | Other perinatal conditions |
| P01.2 | Newborn (suspected to be) affected by oligohydramnios | Other perinatal conditions |
| P01.3 | Newborn (suspected to be) affected by polyhydramnios | Other perinatal conditions |
| P01.4 | Newborn (suspected to be) affected by ectopic pregnancy | Other perinatal conditions |
| P01.5 | Newborn (suspected to be) affected by multiple pregnancy | Other perinatal conditions |
| P01.6 | Newborn (suspected to be) affected by maternal death | Other perinatal conditions |
| P01.7 | Newborn (suspected to be) affected by malpresentation before labor | Other perinatal conditions |
| P01.8 | Newborn (suspected to be) affected by other maternal complications of pregnancy | Other perinatal conditions |
| P01.9 | Newborn (suspected to be) affected by maternal complication of pregnancy, unspecified | Other perinatal conditions |
| P02 | Fetus and newborn affected by complications of placenta, cord and membranes | Other perinatal conditions |
| P02.0 | Newborn (suspected to be) affected by placenta previa | Other perinatal conditions |
| P02.1 | Newborn (suspected to be) affected by other forms of placental separation and hemorrhage | Other perinatal conditions |
| P02.2 | Fetus and newborn affected by other and unspecified morphological and functional abnormalities of placenta. | Other perinatal conditions |
| P02.3 | Newborn (suspected to be) affected by placental transfusion syndromes | Other perinatal conditions |
| P02.4 | Newborn (suspected to be) affected by prolapsed cord | Other perinatal conditions |
| P02.5 | Newborn (suspected to be) affected by other compression of umbilical cord | Other perinatal conditions |
| P02.6 | Fetus and newborn affected by other and unspecified conditions of umbilical cord | Other perinatal conditions |
| P02.7 | Newborn (suspected to be) affected by chorioamnionitis | Other perinatal conditions |
| P02.8 | Newborn (suspected to be) affected by other abnormalities of membranes | Other perinatal conditions |
| P02.9 | Newborn (suspected to be) affected by abnormality of membranes, unspecified | Other perinatal conditions |
| P03 | Fetus and newborn affected by other complications of labour and delivery | Other perinatal conditions |
| P03.0 | Newborn (suspected to be) affected by breech delivery and extraction | Other perinatal conditions |
| P03.1 | Newborn (suspected to be) affected by other malpresentation, malposition and disproportion during labour and delivery | Other perinatal conditions |
| P03.2 | Newborn (suspected to be) affected by forceps delivery | Other perinatal conditions |
| P03.3 | Newborn (suspected to be) affected by delivery by vacuum extractor [ventouse] | Other perinatal conditions |
| P03.4 | Newborn (suspected to be) affected by Cesarean delivery | Other perinatal conditions |
| P03.5 | Newborn (suspected to be) affected by precipitate delivery | Other perinatal conditions |
| P03.6 | Newborn (suspected to be) affected by abnormal uterine contractions | Other perinatal conditions |
| P03.8 | Fetus and newborn affected by other specified complications of labour and delivery | Other perinatal conditions |
| P03.9 | Newborn (suspected to be) affected by complication of labor and delivery, unspecified | Other perinatal conditions |
| P04 | Fetus and newborn affected by noxious influences transmitted via placenta or breast milk | Other perinatal conditions |
| P04.0 | Newborn (suspected to be) affected by maternal anesthesia and analgesia in pregnancy, labor and delivery | Other perinatal conditions |
| P04.1 | Newborn (suspected to be) affected by other maternal medication | Other perinatal conditions |
| P04.2 | Newborn (suspected to be) affected by maternal use of tobacco | Other perinatal conditions |
| P04.3 | Newborn (suspected to be) affected by maternal use of alcohol | Other perinatal conditions |
| P04.4 | Fetus and newborn affected by maternal use of drugs of addiction | Other perinatal conditions |
| P04.5 | Newborn (suspected to be) affected by maternal use of nutritional chemical substances | Other perinatal conditions |
| P04.6 | Newborn (suspected to be) affected by maternal exposure to environmental chemical substances | Other perinatal conditions |
| P04.8 | Newborn (suspected to be) affected by other maternal noxious substances | Other perinatal conditions |
| P04.9 | Newborn (suspected to be) affected by maternal noxious substance, unspecified | Other perinatal conditions |
| P08 | Disorders related to long gestation and high birth weight | Other perinatal conditions |
| P08.0 | Exceptionally large newborn baby | Other perinatal conditions |
| P08.1 | Other heavy for gestational age newborn | Other perinatal conditions |
| P08.2 | Post-term infant, not heavy for gestational age | Other perinatal conditions |
| P22 | Respiratory distress of newborn | Other perinatal conditions |
| P22.1 | Transient tachypnea of newborn | Other perinatal conditions |
| P22.8 | Other respiratory distress of newborn | Other perinatal conditions |
| P22.9 | Respiratory distress of newborn, unspecified | Other perinatal conditions |
| P23 | Congenital pneumonia | Other perinatal conditions |
| P23.0 | Congenital pneumonia due to viral agent | Other perinatal conditions |
| P23.1 | Congenital pneumonia due to Chlamydia | Other perinatal conditions |
| P23.2 | Congenital pneumonia due to staphylococcus | Other perinatal conditions |
| P23.3 | Congenital pneumonia due to streptococcus, group B | Other perinatal conditions |
| P23.4 | Congenital pneumonia due to Escherichia coli | Other perinatal conditions |
| P23.5 | Congenital pneumonia due to Pseudomonas | Other perinatal conditions |
| P23.6 | Congenital pneumonia due to other bacterial agents | Other perinatal conditions |
| P23.8 | Congenital pneumonia due to other organisms | Other perinatal conditions |
| P23.9 | Congenital pneumonia, unspecified | Other perinatal conditions |
| P24 | Neonatal aspiration syndromes | Other perinatal conditions |
| P24.0 | Neonatal aspiration of meconium | Other perinatal conditions |
| P24.1 | Neonatal aspiration of amniotic fluid and mucus | Other perinatal conditions |
| P24.2 | Neonatal aspiration of blood | Other perinatal conditions |
| P24.3 | Neonatal aspiration of milk and regurgitated food | Other perinatal conditions |
| P24.8 | Other neonatal aspiration syndromes | Other perinatal conditions |
| P24.9 | Neonatal aspiration, unspecified | Other perinatal conditions |
| P25 | Interstitial emphysema and related conditions originating in the perinatal period | Other perinatal conditions |
| P25.0 | Interstitial emphysema originating in the perinatal period | Other perinatal conditions |
| P25.1 | Pneumothorax originating in the perinatal period | Other perinatal conditions |
| P25.2 | Pneumomediastinum originating in the perinatal period | Other perinatal conditions |
| P25.3 | Pneumopericardium originating in the perinatal period | Other perinatal conditions |
| P25.8 | Other conditions related to interstitial emphysema originating in the perinatal period | Other perinatal conditions |
| P26 | Pulmonary haemorrhage originating in the perinatal period | Other perinatal conditions |
| P26.0 | Tracheobronchial hemorrhage originating in the perinatal period | Other perinatal conditions |
| P26.1 | Massive pulmonary hemorrhage originating in the perinatal period | Other perinatal conditions |
| P26.8 | Other pulmonary hemorrhages originating in the perinatal period | Other perinatal conditions |
| P26.9 | Unspecified pulmonary hemorrhage originating in the perinatal period | Other perinatal conditions |
| P27 | Chronic respiratory disease originating in the perinatal period | Other perinatal conditions |
| P27 | Chronic respiratory disease originating in the perinatal period | Other perinatal conditions |
| P27.0 | Wilson-Mikity syndrome | Other perinatal conditions |
| P27.0 | Wilson-Mikity syndrome | Other perinatal conditions |
| P27.1 | Bronchopulmonary dysplasia originating in the perinatal period | Other perinatal conditions |
| P27.1 | Bronchopulmonary dysplasia originating in the perinatal period | Other perinatal conditions |
| P27.8 | Other chronic respiratory diseases originating in the perinatal period | Other perinatal conditions |
| P27.8 | Other chronic respiratory diseases originating in the perinatal period | Other perinatal conditions |
| P27.9 | Unspecified chronic respiratory disease originating in the perinatal period | Other perinatal conditions |
| P27.9 | Unspecified chronic respiratory disease originating in the perinatal period | Other perinatal conditions |
| P28 | Other respiratory conditions originating in the perinatal period | Other perinatal conditions |
| P28 | Other respiratory conditions originating in the perinatal period | Other perinatal conditions |
| P28.0 | Primary atelectasis of newborn | Other perinatal conditions |
| P28.0 | Primary atelectasis of newborn | Other perinatal conditions |
| P28.1 | Other and unspecified atelectasis of newborn | Other perinatal conditions |
| P28.1 | Other and unspecified atelectasis of newborn | Other perinatal conditions |
| P28.2 | Cyanotic attacks of newborn | Other perinatal conditions |
| P28.2 | Cyanotic attacks of newborn | Other perinatal conditions |
| P28.3 | Primary sleep apnea of newborn | Other perinatal conditions |
| P28.3 | Primary sleep apnea of newborn | Other perinatal conditions |
| P28.4 | Other apnea of newborn | Other perinatal conditions |
| P28.4 | Other apnea of newborn | Other perinatal conditions |
| P28.5 | Respiratory failure of newborn | Other perinatal conditions |
| P28.5 | Respiratory failure of newborn | Other perinatal conditions |
| P28.8 | Other specified respiratory conditions of newborn | Other perinatal conditions |
| P28.8 | Other specified respiratory conditions of newborn | Other perinatal conditions |
| P28.9 | Respiratory condition of newborn, unspecified | Other perinatal conditions |
| P28.9 | Respiratory condition of newborn, unspecified | Other perinatal conditions |
| P29 | Cardiovascular disorders originating in the perinatal period | Other perinatal conditions |
| P29.0 | Neonatal cardiac failure | Other perinatal conditions |
| P29.1 | Neonatal cardiac dysrhythmia | Other perinatal conditions |
| P29.2 | Neonatal hypertension | Other perinatal conditions |
| P29.4 | Transient myocardial ischemia in newborn | Other perinatal conditions |
| P29.8 | Other cardiovascular disorders originating in the perinatal period | Other perinatal conditions |
| P29.9 | Cardiovascular disorder originating in the perinatal period, unspecified | Other perinatal conditions |
| P35 | Congenital viral diseases | Other perinatal conditions |
| P35.0 | Congenital rubella syndrome | Other perinatal conditions |
| P35.1 | Congenital cytomegalovirus infection | Other perinatal conditions |
| P35.8 | Other congenital viral diseases | Other perinatal conditions |
| P35.9 | Congenital viral disease, unspecified | Other perinatal conditions |
| P36 | Bacterial sepsis of newborn | Other perinatal conditions |
| P36.0 | Sepsis of newborn due to streptococcus, group B | Other perinatal conditions |
| P36.1 | Sepsis of newborn due to other and unspecified streptococci | Other perinatal conditions |
| P36.2 | Sepsis of newborn due to Staphylococcus aureus | Other perinatal conditions |
| P36.3 | Sepsis of newborn due to other and unspecified staphylococci | Other perinatal conditions |
| P36.4 | Sepsis of newborn due to Escherichia coli | Other perinatal conditions |
| P36.5 | Sepsis of newborn due to anaerobes | Other perinatal conditions |
| P36.8 | Other bacterial sepsis of newborn | Other perinatal conditions |
| P36.9 | Bacterial sepsis of newborn, unspecified | Other perinatal conditions |
| P37 | Other congenital infectious and parasitic diseases | Other perinatal conditions |
| P37.1 | Congenital toxoplasmosis | Other perinatal conditions |
| P37.2 | Neonatal (disseminated) listeriosis | Other perinatal conditions |
| P37.3 | Congenital falciparum malaria | Other perinatal conditions |
| P37.4 | Other congenital malaria | Other perinatal conditions |
| P37.5 | Neonatal candidiasis | Other perinatal conditions |
| P37.8 | Other specified congenital infectious and parasitic diseases | Other perinatal conditions |
| P37.9 | Congenital infectious or parasitic disease, unspecified | Other perinatal conditions |
| P38 | Omphalitis of newborn with or without mild haemorrhage | Other perinatal conditions |
| P39 | Other infections specific to the perinatal period | Other perinatal conditions |
| P39.0 | Neonatal infective mastitis | Other perinatal conditions |
| P39.1 | Neonatal conjunctivitis and dacryocystitis | Other perinatal conditions |
| P39.2 | Intra-amniotic infection affecting newborn, not elsewhere classified | Other perinatal conditions |
| P39.4 | Neonatal skin infection | Other perinatal conditions |
| P39.8 | Other specified infections specific to the perinatal period | Other perinatal conditions |
| P39.9 | Infection specific to the perinatal period, unspecified | Other perinatal conditions |
| P50 | Fetal blood loss | Other perinatal conditions |
| P50.0 | Newborn affected by intrauterine (fetal) blood loss from vasa previa | Other perinatal conditions |
| P50.1 | Newborn affected by intrauterine (fetal) blood loss from ruptured cord | Other perinatal conditions |
| P50.2 | Newborn affected by intrauterine (fetal) blood loss from placenta | Other perinatal conditions |
| P50.3 | Newborn affected by hemorrhage into co-twin | Other perinatal conditions |
| P50.4 | Newborn affected by hemorrhage into maternal circulation | Other perinatal conditions |
| P50.5 | Newborn affected by intrauterine (fetal) blood loss from cut end of co-twins cord | Other perinatal conditions |
| P50.8 | Newborn affected by other intrauterine (fetal) blood loss | Other perinatal conditions |
| P50.9 | Newborn affected by intrauterine (fetal) blood loss, unspecified | Other perinatal conditions |
| P51 | Umbilical haemorrhage of newborn | Other perinatal conditions |
| P51.0 | Massive umbilical hemorrhage of newborn | Other perinatal conditions |
| P51.8 | Other umbilical hemorrhages of newborn | Other perinatal conditions |
| P51.9 | Umbilical hemorrhage of newborn, unspecified | Other perinatal conditions |
| P52 | Intracranial nontraumatic haemorrhage of fetus and newborn | Other perinatal conditions |
| P52.0 | Intraventricular (nontraumatic) hemorrhage, grade 1, of newborn | Other perinatal conditions |
| P52.1 | Intraventricular (nontraumatic) hemorrhage, grade 2, of newborn | Other perinatal conditions |
| P52.2 | Intraventricular (nontraumatic) haemorrhage, grade 3, of fetus and newborn | Other perinatal conditions |
| P52.3 | Unspecified intraventricular (nontraumatic) hemorrhage of newborn | Other perinatal conditions |
| P52.4 | Intracerebral (nontraumatic) hemorrhage of newborn | Other perinatal conditions |
| P52.5 | Subarachnoid (nontraumatic) hemorrhage of newborn | Other perinatal conditions |
| P52.6 | Cerebellar (nontraumatic) and posterior fossa hemorrhage of newborn | Other perinatal conditions |
| P52.8 | Other intracranial (nontraumatic) hemorrhages and newborn | Other perinatal conditions |
| P52.9 | Intracranial (nontraumatic) hemorrhage of newborn, unspecified | Other perinatal conditions |
| P53 | Hemorrhagic disease of newborn | Other perinatal conditions |
| P54 | Other neonatal haemorrhages | Other perinatal conditions |
| P54.0 | Neonatal hematemesis | Other perinatal conditions |
| P54.1 | Neonatal melena | Other perinatal conditions |
| P54.2 | Neonatal rectal hemorrhage | Other perinatal conditions |
| P54.3 | Other neonatal gastrointestinal hemorrhage | Other perinatal conditions |
| P54.4 | Neonatal adrenal hemorrhage | Other perinatal conditions |
| P54.5 | Neonatal cutaneous hemorrhage | Other perinatal conditions |
| P60 | Disseminated intravascular coagulation of newborn | Other perinatal conditions |
| P61 | Other perinatal haematological disorders | Other perinatal conditions |
| P61.0 | Transient neonatal thrombocytopenia | Other perinatal conditions |
| P61.1 | Polycythemia neonatorum | Other perinatal conditions |
| P61.2 | Anemia of prematurity | Other perinatal conditions |
| P61.3 | Congenital anemia from fetal blood loss | Other perinatal conditions |
| P61.4 | Other congenital anemias, not elsewhere classified | Other perinatal conditions |
| P61.5 | Transient neonatal neutropenia | Other perinatal conditions |
| P61.6 | Other transient neonatal disorders of coagulation | Other perinatal conditions |
| P61.8 | Other specified perinatal hematological disorders | Other perinatal conditions |
| P61.9 | Perinatal hematological disorder, unspecified | Other perinatal conditions |
| P70 | Transitory disorders of carbohydrate metabolism specific to fetus and newborn | Other perinatal conditions |
| P70.0 | Syndrome of infant of mother with gestational diabetes | Other perinatal conditions |
| P70.1 | Syndrome of infant of a diabetic mother | Other perinatal conditions |
| P70.2 | Neonatal diabetes mellitus | Other perinatal conditions |
| P70.3 | Iatrogenic neonatal hypoglycemia | Other perinatal conditions |
| P70.4 | Other neonatal hypoglycemia | Other perinatal conditions |
| P70.8 | Other transitory disorders of carbohydrate metabolism of newborn | Other perinatal conditions |
| P70.9 | Transitory disorder of carbohydrate metabolism of newborn, unspecified | Other perinatal conditions |
| P71 | Transitory neonatal disorders of calcium and magnesium metabolism | Other perinatal conditions |
| P71.0 | Cows milk hypocalcemia in newborn | Other perinatal conditions |
| P71.1 | Other neonatal hypocalcemia | Other perinatal conditions |
| P71.2 | Neonatal hypomagnesemia | Other perinatal conditions |
| P71.3 | Neonatal tetany without calcium or magnesium deficiency | Other perinatal conditions |
| P71.4 | Transitory neonatal hypoparathyroidism | Other perinatal conditions |
| P71.8 | Other transitory neonatal disorders of calcium and magnesium metabolism | Other perinatal conditions |
| P71.9 | Transitory neonatal disorder of calcium and magnesium metabolism, unspecified | Other perinatal conditions |
| P72 | Other transitory neonatal endocrine disorders | Other perinatal conditions |
| P72.2 | Other transitory neonatal disorders of thyroid function, not elsewhere classified | Other perinatal conditions |
| P72.8 | Other specified transitory neonatal endocrine disorders | Other perinatal conditions |
| P72.9 | Transitory neonatal endocrine disorder, unspecified | Other perinatal conditions |
| P74 | Other transitory neonatal electrolyte and metabolic disturbances | Other perinatal conditions |
| P74.0 | Late metabolic acidosis of newborn | Other perinatal conditions |
| P74.1 | Dehydration of newborn | Other perinatal conditions |
| P74.2 | Disturbances of sodium balance of newborn | Other perinatal conditions |
| P74.3 | Disturbances of potassium balance of newborn | Other perinatal conditions |
| P74.4 | Other transitory electrolyte disturbances of newborn | Other perinatal conditions |
| P74.5 | Transitory tyrosinemia of newborn | Other perinatal conditions |
| P74.8 | Other transitory metabolic disturbances of newborn | Other perinatal conditions |
| P74.9 | Transitory metabolic disturbance of newborn, unspecified | Other perinatal conditions |
| P75* | Meconium ileusin cystic fibrosis (E84.1+) | Other perinatal conditions |
| P76 | Other intestinal obstruction of newborn | Other perinatal conditions |
| P76.0 | Meconium plug syndrome | Other perinatal conditions |
| P76.1 | Transitory ileus of newborn | Other perinatal conditions |
| P76.2 | Intestinal obstruction due to inspissated milk | Other perinatal conditions |
| P76.8 | Other specified intestinal obstruction of newborn | Other perinatal conditions |
| P76.9 | Intestinal obstruction of newborn, unspecified | Other perinatal conditions |
| P77 | Necrotizing enterocolitis of newborn | Other perinatal conditions |
| P78 | Other perinatal digestive system disorders | Other perinatal conditions |
| P78.0 | Perinatal intestinal perforation | Other perinatal conditions |
| P78.1 | Other neonatal peritonitis | Other perinatal conditions |
| P78.2 | Neonatal hematemesis and melena due to swallowed maternal blood | Other perinatal conditions |
| P78.3 | Noninfective neonatal diarrhea | Other perinatal conditions |
| P78.8 | Other specified perinatal digestive system disorders | Other perinatal conditions |
| P78.9 | Perinatal digestive system disorder, unspecified | Other perinatal conditions |
| P80 | Hypothermia of newborn | Other perinatal conditions |
| P80.0 | Cold injury syndrome | Other perinatal conditions |
| P80.8 | Other hypothermia of newborn | Other perinatal conditions |
| P80.9 | Hypothermia of newborn, unspecified | Other perinatal conditions |
| P81 | Other disturbances of temperature regulation of newborn | Other perinatal conditions |
| P81.0 | Environmental hyperthermia of newborn | Other perinatal conditions |
| P81.8 | Other specified disturbances of temperature regulation of newborn | Other perinatal conditions |
| P81.9 | Disturbance of temperature regulation of newborn, unspecified | Other perinatal conditions |
| P83 | Other conditions of integument specific to fetus and newborn | Other perinatal conditions |
| P83.0 | Sclerema neonatorum | Other perinatal conditions |
| P83.1 | Neonatal erythema toxicum | Other perinatal conditions |
| P83.2 | Hydrops fetalis not due to hemolytic disease | Other perinatal conditions |
| P83.3 | Other and unspecified oedema specific to fetus and newborn | Other perinatal conditions |
| P83.4 | Breast engorgement of newborn | Other perinatal conditions |
| P83.5 | Congenital hydrocele | Other perinatal conditions |
| P83.6 | Umbilical polyp of newborn | Other perinatal conditions |
| P83.8 | Other specified conditions integument specific to newborn | Other perinatal conditions |
| P83.9 | Condition of the integument specific to newborn, unspecified | Other perinatal conditions |
| P90 | Convulsions of newborn | Other perinatal conditions |
| P91 | Other disturbances of cerebral status of newborn | Other perinatal conditions |
| P91.0 | Neonatal cerebral ischemia | Other perinatal conditions |
| P91.1 | Acquired periventricular cysts of newborn | Other perinatal conditions |
| P91.2 | Neonatal cerebral leukomalacia | Other perinatal conditions |
| P91.3 | Neonatal cerebral irritability | Other perinatal conditions |
| P91.4 | Neonatal cerebral depression | Other perinatal conditions |
| P91.5 | Neonatal coma | Other perinatal conditions |
| P91.6 | Hypoxic ischemic encephalopathy (HIE) | Other perinatal conditions |
| P91.8 | Other specified disturbances of cerebral status of newborn | Other perinatal conditions |
| P91.9 | Disturbance of cerebral status of newborn, unspecified | Other perinatal conditions |
| P92 | Feeding problems of newborn | Other perinatal conditions |
| P92.0 | Vomiting of newborn | Other perinatal conditions |
| P92.1 | Regurgitation and rumination of newborn | Other perinatal conditions |
| P92.2 | Slow feeding of newborn | Other perinatal conditions |
| P92.3 | Underfeeding of newborn | Other perinatal conditions |
| P92.4 | Overfeeding of newborn | Other perinatal conditions |
| P92.5 | Neonatal difficulty in feeding at breast | Other perinatal conditions |
| P92.8 | Other feeding problems of newborn | Other perinatal conditions |
| P92.9 | Feeding problem of newborn, unspecified | Other perinatal conditions |
| P93 | Reactions and intoxications due to drugs administered to fetus and newborn | Other perinatal conditions |
| P94 | Disorders of muscle tone of newborn | Other perinatal conditions |
| P94.0 | Transient neonatal myasthenia gravis | Other perinatal conditions |
| P94.1 | Congenital hypertonia | Other perinatal conditions |
| P94.2 | Congenital hypotonia | Other perinatal conditions |
| P94.8 | Other disorders of muscle tone of newborn | Other perinatal conditions |
| P94.9 | Disorder of muscle tone of newborn, unspecified | Other perinatal conditions |
| P96 | Other conditions originating in the perinatal period | Other perinatal conditions |
| P96.0 | Congenital renal failure | Other perinatal conditions |
| P96.1 | Neonatal withdrawal symptoms from maternal use of drugs of addiction | Other perinatal conditions |
| P96.4 | Termination of pregnancy, fetus and newborn | Other perinatal conditions |
| P96.5 | Complication to newborn due to (fetal) intrauterine procedure | Other perinatal conditions |
| P96.8 | Other specified conditions originating in the perinatal period | Other perinatal conditions |
| P96.9 | Condition originating in the perinatal period, unspecified | Other perinatal conditions |
| G80 | Cerebral palsy |  |
| G80.0 | Spastic quadriplegic cerebral palsy |  |
| G80.1 | Spastic diplegic cerebral palsy |  |
| G80.2 | Spastic hemiplegic cerebral palsy |  |
| G80.3 | Athetoid cerebral palsy |  |
| G80.4 | Ataxic cerebral palsy |  |
| G80.8 | Other cerebral palsy |  |
| G80.9 | Cerebral palsy, unspecified |  |

Table S5: Covariates and outcomes in children with full vs censored follow up

|  | **Registered throughout** | **Deregistered*** | **Total** |  |
| --- | --- | --- | --- | --- |
| **Total** | n=221,944 | n=97,836 | n=319,780 |  |
|  | No. (%) | No. (%) | No. | χ^2^ test p value |
| ***Index of Multiple Deprivation*** |  |  |  |  |
| **1: Least deprived** | 54,252 (24·4) | 19,981 (20·4) | 74,233 (23·2) |  |
| **2** | 47,569 (21·4) | 19,006 (19·4) | 66,575 (20·8) |  |
| **3** | 41,383 (18·7) | 18,172 (18·6) | 59,555 (18·6) |  |
| **4** | 41,245 (18·6) | 20,732 (21·2) | 61,977 (19·4) |  |
| **5: Most deprived** | 37,495 (16·9) | 19,945 (20·4) | 57,440 (18·0) | p<0·001 |
| ***Maternal Age (at delivery)*** |  |  |  |  |
| **<20** | 7,045 (3·2) | 5,769 (5·9) | 12,814 (4·0) |  |
| **20-<45** | 203,143 (91·5) | 88,703 (90·7) | 291,846 (91·3) |  |
| **45+** | 11,756 (5·3) | 3,364 (3·4) | 15,120 (4·7) | p<0·001 |
| ***Sex*** |  |  |  |  |
| **Boys** | 113,645 (51·2) | 50,068 (51·2) | 163,713 (51·2) |  |
| **Girls** | 108,299 (48·8) | 47,768 (48·8) | 156,067 (48·8) | p= 0·880 |
| **Vaccination** |  |  |  |  |
| **<3 vaccines** | 8,450 (3·8) | 13,929 (14·2) | 22,379 (7·0) |  |
| **3+ vaccines** | 213,494 (96·2) | 83,907 (85·8) | 297,401 (93·0) | p<0·001 |
| ***Development checks*** |  |  |  |  |
| **0** | 28,347 (12·8) | 17,348 (17·7) | 45,695 (14·3) |  |
| **1+** | 193,597 (87·2) | 80,488 (82·3) | 274,085 (85·7) | p<0·001 |
| ***Health service use in first year*** | Mean (95% CI) | Mean (95% CI) | Mean (95% CI) | T test p value |
| **Unplanned admission rates** | 171  (169 to 172) | 179  (177 to 181) | 173  (172 to 175) | p<0·001 |
| **Illness consultation rates** | 4·10  (4·08 to 4·11) | 3·80  (3·77 to 3·82) | 4·01  (3·99 to 4·02) | p<0·001 |
| * Children who deregistered from their practice during follow up | | | | |

Table S6: Adjusted hazard ratios for unplanned admission stratified by full vs censored follow up, in infants

|  |  | **Infant (Aged <1 year)** | |
| --- | --- | --- | --- |
|  |  | **Full fu^a^** | **Censored during fu^a^** |
|  |  | **HR^b^ (95% CI)** | **HR^b^ (95% CI)** |
| **IMD^c^** | 5 vs 1 | 1·32 (1·28 to 1·36) | 1·44 (1·38 to 1·51) |
| **Illness consultation rate** | | 1·005 (1·005 to 1·005) | 1·019 (1·018 to 1·019) |
| **Sex** | Girl vs boy | 0·81 (0·79 to 0·82) | 0·80 (0·78 to 0·83) |
| **Maternal age** | <20yrs vs 20-39 | 1·34 (1·28 to 1·41) | 1·41 (1·34 to 1·49) |
|  | 40+ yrs vs 20-39 | 0·92 (0·88 to 0·97) | 0·78 (0·71 to 0·86) |
| a) fu – follow-up; b) Hazard ratio and 95% confidence interval; c) Indices multiple deprivation fifths, 5 is the most deprived and 1 the least deprived. | | | |

**Table S7: Association of preventive primary care, comorbidity and social factors on risk of unplanned hospital admission in using a random intercept model clustering by GP practice**

|  | Hazard Ratio (95% CI) | | |
| --- | --- | --- | --- |
|  | **Aged <1 year** | **Aged 1-4 year** | **Aged 5-9 year** |
| Incomplete vaccinations | 1.84 (1.79 to 1.89) | 1.91 (1.81 to 2.02) | 1.24 (1.04 to 1.48) |
| No development checks | 6.94 (6.81 to 7.08) | 1.22 (1.19 to 1.26) | 1.10 (1.04 to 1.17) |
| Illness consultation rate | 1.02 (1.02 to 1.02) | 1.19 (1.19 to 1.19) | 1.28 (1.27 to 1.29) |
| Preterm birth | 1.11 (1.08 to 1.15) | 1.24 (1.20 to 1.28) | 1.11 (1.03 to 1.19) |
| Congenital conditions | 2.36 (2.31 to 2.40) | 1.17 (1.15 to 1.20) | 1.10 (1.05 to 1.16) |
| Deprivation  (most vs least deprived) | 1.11 (1.07 to 1.14) | 1.14 (1.11 to 1.18) | 1.24 (1.16 to 1.32) |
| Maternal age |  |  |  |
| <20 vs 20-39 | 1.23 (1.18 to 1.28) | 1.28 (1.23 to 1.33) | 1.23 (1.13 to 1.33) |
| 40+ vs 20-39 | 0.91 (0.87 to 0.95) | 0.98 (0.94 to 1.02) | 1.00 (0.91 to 1.09) |
| Being first born | 0.89 (0.87 to 0.90) | 0.97 (0.96 to 0.99) |  |
| a) Hazard ratios have been adjusted for sex and all listed covariates. b) face to face consultation with a GP excluding preventive care; c) Less than 3 infant vaccinations; d) IMD-Index of multiple deprivation fifths (5 the most deprived, 1 least deprived). Cohort of children born between 01/01/2000 and 31/03/2013 registered with 363 practices partnered with the Clinical Practice Research Data link-CPRD linked to Hospital Episode Statistics in England and followed up until 31/12/2013. | | | |
