# Supplementary material for: Impact of preventive primary care on children’s unplanned hospital admissions: a population-based birth cohort study of UK children 2000–2013
Source: BMC Med. 2018 Sep 17;16:151. doi: 10.1186/s12916-018-1142-3 (PMC6139908; doi:10.1186/s12916-018-1142-3)
Supplement: Supplementary file 2 — A supplementary file detailing the methodology used for creating the birth cohort; the preventive care consultations and illness consultations. (DOCX 24 kb) [file 12916_2018_1142_MOESM2_ESM.docx]

**Supplementary Methods**

# Creating a birth cohort

The Clinical Practice Research Datalink (CPRD) provides two sets of data quality criteria: acceptability for patients and up to standard (UTS) time for practices. We used these criteria in developing the birth cohort. Our target population was children born between 1^st^ January 2000 and 31^st^ March 2013 and we used the following procedure:

1. Using CPRD ‘acceptable’ patient table, we isolated children (with a birth year of 2000 through to 2012 or a birth year of 2013 and a birth month of January, February and March).
2. We selected those children whose health record was linked (via the child’s CPRD Id) to a mother using CPRD’s Mother and Baby link table. The reason for the linkage was to firstly to gather basic information on the mother but also to secure a full consulting history for each child from birth. Our assumption was that children do not have any health contacts with other GPs prior to their registration at the CPRD-participating practice to which their mother is registered. This link also provided us with a mother’s delivery date for the child.
3. We excluded children if their mother, at time of the child’s birth, was not registered with the child’s (later) registered practice.
4. We excluded children if the registered CPRD-participating practice was not ‘up to standard’ at time of the child’s birth.
5. We excluded children without HES linkage
   HES data provides the outcome but also children born into an NHS hospital should have a record of their birth.
6. Children entered the cohort at birth. The date of birth was taken for the HES records. These were records with an admission method of:
   82 The birth of a baby in the recorded Health Care Provider
7. Baby born outside the Health Care Provider except when born at home as intended.

18% of children did not have a HES birth record. There may be several reasons for this including children born at home or children of a difficult birth where the admission method was recorded as an emergency.

95% of birth dates in the HES data was within 1 month of the delivery date recorded in CPRD; 99.5% within 2 months.

1. Children who died over the follow-up period were excluded from the analysis.
   CPRD - a recorded death date

HES – a discharge method of ‘died’

# Creating preventive consultations

## Development checks

Using CPRD clinical data, we flagged consultations, in infants, where there was a recording of a development check (Read codes 64… and ZV202).

## Vaccinations

Using CPRD immunization data, we identified consultations in infants for vaccinations.

# Creating GP consultations for illness

Using CPRD clinical data we:

1. Defined a GP consultation as direct contact between a GP and a child using the consultation type look up table COT. Direct consultations were for Clinic, Follow-up/routine visit, Surgery consultation, Acute visit and Emergency Consultation.
2. Differentiated between consultation with a GP and consultation with another practice member of staff by highlighting the staff who were GPs using the staff id and the Role of staff look up table ROL.
3. Excluded records that were administrative using the Consultation type look up table SED
4. We excluded GP consultations for preventive care (using the patient id and consultation ids isolated in preventive care records above).

This process is laid out in Supplementary Figure 1.

Supplementary Figure 1: Process for determining a GP consultation for illness
